# Urinary metabolites predict mortality or need for renal replacement therapy after combat injury

**DOI:** 10.1186/s13054-021-03544-2

**Published:** 2021-03-23

**Authors:** Sarah Gisewhite, Ian J. Stewart, Greg Beilman, Elizabeth Lusczek

**Affiliations:** 1 Department of Surgery, University of Minnesota, 515 Delaware St SE, Minneapolis, MN 55455 USA; 2 Department of Medicine, Uniformed Services University, 4301 Jones Bridge Road, Bethesda, MD 20814 USA

**Keywords:** Acute kidney injury, Biomarkers, Metabolites, Combat injury, Risk prediction, Metabolomics, Renal replacement therapy

## Abstract

**Background:**

Traditionally, patient risk scoring is done by evaluating vital signs and clinical severity scores with clinical intuition. Urinary biomarkers can add objectivity to these models to make risk prediction more accurate. We used metabolomics to identify prognostic urinary biomarkers of mortality or need for renal replacement therapy (RRT). Additionally, we assessed acute kidney injury (AKI) diagnosis, injury severity score (ISS), and AKI stage.

**Methods:**

Urine samples (*n* = 82) from a previous study of combat casualties were evaluated using proton nuclear magnetic resonance (^1^H-NMR) spectroscopy. Chenomx software was used to identify and quantify urinary metabolites. Metabolite concentrations were normalized by urine output, autoscaled, and log-transformed. Partial least squares discriminant analysis (PLS-DA) and statistical analysis were performed. Receiver operating characteristic (ROC) curves were used to assess prognostic utility of biomarkers for mortality and RRT.

**Results:**

Eighty-four (84) metabolites were identified and quantified in each urine sample. Of these, 11 were identified as drugs or drug metabolites and excluded. The PLS-DA models for ISS and AKI diagnosis did not have acceptable model statistics. Therefore, only mortality/RRT and AKI stage were analyzed further. Of 73 analyzed metabolites, 9 were significantly associated with mortality/RRT (*p* < 0.05) and 11 were significantly associated with AKI stage (*p* < 0.05). 1-Methylnicotinamide was the only metabolite to be significantly associated (*p* < 0.05) with all outcomes and was significantly higher (*p* < 0.05) in patients with adverse outcomes. Elevated lactate and 1-methylnicotinamide levels were associated with higher AKI stage and mortality and RRT, whereas elevated glycine levels were associated with patients who survived and did not require RRT, or had less severe AKI. ROC curves for each of these metabolites and the combined panel had good predictive value (lactate AUC = 0.901, 1-methylnicotinamide AUC = 0.864, glycine AUC = 0.735, panel AUC = 0.858).

**Conclusions:**

We identified urinary metabolites associated with AKI stage and the primary outcome of mortality or need for RRT. Lactate, 1-methylnicotinamide, and glycine may be used as a panel of predictive biomarkers for mortality and RRT. 1-Methylnicotinamide is a novel biomarker associated with adverse outcomes. Additional studies are necessary to determine how these metabolites can be utilized in clinically-relevant risk prediction models.

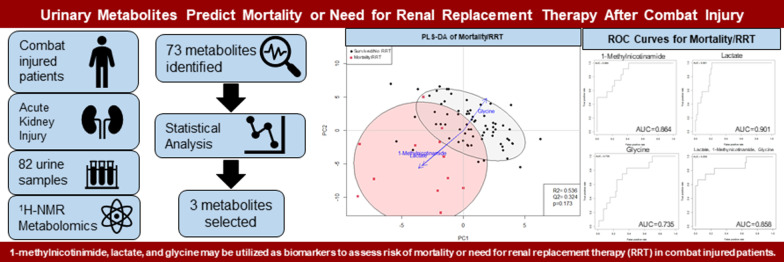

**Supplementary Information:**

The online version contains supplementary material available at 10.1186/s13054-021-03544-2.

## Background

Traditionally, patient risk scoring is done by evaluating vital signs and clinical severity scores with clinical intuition [1]. Risk prediction is especially important in hospitals caring for combat injured patients. This patient population may have delayed access to care, limited access to resources, multiple mechanisms of injury, more severe injury, and higher risk of a penetrating injury [2]. Accurate prediction models may allow for better resource allocation, more efficient triage of patients, and identify patients at higher risk for an adverse outcome, which will improve patient survival and recovery [1].

In addition, organ dysfunction, such as acute kidney injury (AKI), can cause complications in critically ill patients and increase the risk of mortality [3]. Acute kidney injury (AKI) is defined as a sudden decrease of kidney function caused by direct kidney injury or functional impairment [4]. Among intensive care unit (ICU) patients with AKI, mortality is around 50%, and in patients that require renal replacement therapy (RRT) it can be as high as 80% [3]. Important barriers to improving AKI treatment and outcomes are identifying patients at risk for non-recovery or developing severe AKI [5].

To this end, we explored urinary biomarkers that could predict mortality and the need for RRT (which is severe AKI). Urinary biomarkers can add objectivity to risk prediction models to make risk prediction more accurate [1]. Additionally, we investigated biomarkers of AKI diagnosis, injury severity score (ISS), and AKI stage. Previous work has been done to evaluate urinary biomarkers associated with AKI, but these studies have focused on a few proteins whose diagnostic ability has been questioned [6]. Due to its complex pathophysiology, it may be necessary to create a panel of biomarkers for diagnostic and prognostic assessment of AKI. Therefore, for the identification and quantification of metabolites, we used metabolomics. An advantage of metabolomics over proteomics and genomics is that metabolomics directly captures phenotypic changes that occur in the system [7].

In this study we used nuclear magnetic resonance (NMR) metabolomics to detect changes in urinary metabolite levels in combat casualties. Specifically, we aimed to identify urinary biomarkers predictive of mortality or need for renal replacement therapy, and biomarkers associated with AKI diagnosis, injury severity score, and AKI severity.

## Materials and methods

### Urine sample collection and patient enrollment

We performed a secondary analysis on urine samples from a published study on risk prediction for combat casualties by Stewart et al. [1]. Of note, the original study included 89 subjects. However, urine from 82 subjects was available for this secondary analysis. Materials and methods for enrollment criteria and consent, sample collection, and IRB approval for human patients (the US Army Medical Research and Materiel Command) have been described [1]. Briefly, patients were US military personnel with traumatic injuries admitted to an ICU in a combat hospital in Afghanistan from October 2012 to December 2013. Patients were excluded if it had been more than 48 h since injury or they did not have a Foley catheter. Urine samples were collected within 3 h of admission, centrifuged at 2000*g* for 10 min, then frozen (− 80 °C) for transport within 1 h of collection [1]. Demographics, injury severity score, blood transfusions and other lab values were collected prospectively. Need for renal replacement therapy and mortality were collected retrospectively. Kidney Disease: Improving Global Outcomes (KDIGO) criteria was used to diagnose and stage AKI [1, 8].

### Urine sample preparation

Urine samples were stored at -80 °C until analysis [1]. Samples were filtered to remove protein with Centrifree Ultrafiltration devices (Millipore, Bilerica, MA). Filters were washed 5 times to remove glycerol. Samples were filtered by centrifugation at 6000 rpm for 2–3 h at 4 °C. For NMR analysis, 1 mL of filtrate was mixed with 0.5 mL of 0.2 M sodium phosphate buffer. The solution was placed on ice for 10 min and then centrifuged at 7000 g at room temperature for 10 min. 500 μL of the supernatant was withdrawn and combined with 50 μL of 1 mM 3-(trimethylsilyl) propionic acid (TSP, Sigma-Aldrich, St. Louis, MO, USA) [9]. The pH of the solution was measured, then the solution was transferred to 5 mm NMR tubes for analysis.

### ^1^H-NMR spectroscopy

One-dimensional proton NMR (^1^H-NMR) spectra were acquired on a 700-MHz Bruker Avance NMR spectrometer with a 5-mm TXI proton-enhanced cryoprobe running TopSpin v. 2.16 (Bruker, Bilerica, MA, USA). A 1D NOESY (Nuclear Overhauser Effect Spectroscopy) pulse sequence was used to collect spectra of each sample. All spectra were collected at 298 K (K).

### Untargeted analysis of spectra

Spectra were analyzed using Chenomx software (Edmonton, AB, Canada) [10]. Manual phasing, baseline correction, and untargeted metabolic profiling using the Chenomx library was performed by a single person. This resulted in a list of identified metabolites and their concentrations in millimoles per liter (mM).

### Normalization

Metabolite concentrations were normalized by urine output (UO) to account for dilution effects in the urine [11]. The urine output used to normalize the data was the average of two hours of urine output [1]. The final units for the normalized metabolite concentrations were mM/h/kg. The conversion and normalization calculations were done in Microsoft Excel (Microsoft, Redmond, WA, USA).

### Statistical analysis

The normalized metabolite concentrations were auto-scaled and log-transformed using R software (R Foundation for Statistical Computing, http://cran.r-proiect.org/) [12]. Partial least squares discriminant analysis (PLS-DA) was performed on the following outcomes (groups): AKI stage (0–1 (none-mild) or 2–3 (moderate-severe)), mortality or required RRT (yes or no), AKI diagnosis (yes or no), injury severity score (< 25 or ≥ 25). Mortality and need for renal replacement therapy were analyzed as a combined endpoint (mortality/RRT) due to small n. Of note, mortality is defined as all-cause mortality with or without AKI. We used the following guidelines to assess our PLS-DA models: for *R*^2^, a value of greater than 0.67 represented high predictive accuracy, 0.33–0.67 was moderate, 0.19–0.33 was low, and below 0.19 was unacceptable. *Q*^2^ values above zero indicated the model had predictive relevance whereas values below zero indicated no predictive relevance, and the *Q*^2^ value should be less than the *R*^2^ value [13]. Permutation *p* values were used to judge the statistical significance of the PLS-DA model. The DiscriMiner package in R was used for the PLS-DA analysis.

Variable importance projection (VIP) scores were used to identify a metabolite’s contribution to the separation between groups in the PLS-DA model. The Wilcoxon rank-sum test was used to evaluate the statistical significance (*p* < 0.05) between the metabolite concentrations of the outcome groups. Boxplots were used to illustrate differences between groups. To determine the diagnostic ability of biomarkers for mortality/RRT, receiver operating characteristic (ROC) curves were made and the area under the curve (AUC) was evaluated. ROC curves were made using the ROCR package in R [14].

## Results

### Sample collection and ^1^H-NMR data

The study population was comprised of 82 patients (US military personnel) admitted to a combat hospital in Afghanistan with traumatic injury requiring ICU-level care [1]. The study population was 96.3% male with an average age of 26.8 ± 5.1 years (Table 1). Compared to patients who survived or did not need RRT, those in the mortality/RRT group had more severe injury indicated by the significantly (*p* < 0.05) higher median lactate levels, median injury severity scores, and percentage of patients requiring a massive transfusion (Table 1).

**Table 1:** Patient cohort characteristics

Characteristic	Full cohort	Survived or No RRT	Mortality or RRT	*p* value
Number	82	70	12	–
Age (years)	26.8 ± 5.1	26.6 ± 5.2	27.8 ± 4.9	0.31
Male (%)	96.3	95.7	100	1.0
Median ISS (IQR)	18.5 (11.8, 40.3)	18 (9, 33.8)	46 (29.3, 63)	0.002
Median lactate (IQR)	1.8 (1.1, 2.9)	1.5 (1, 2.5)	4.3 (2.3, 6.4)	< 0.001
Mass. transfusion (*n* (%))	32 (39)	23 (32.9)	9 (75)	0.009
AKI Diagnosis (*n* (%))	33 (40.2)	23 (32.9)	10 (83.3)	0.003
RRT (*n* (%))	6 (7.3)	–	6 (50)	–
Mortality (*n* (%))	9 (11)	–	9 (75)	–
RRT and survived (*n* (%))	3 (3.7)	–	3 (25)	–
Mortality and no RRT (*n* (%))	6 (7.3)	–	6 (50)	–
No AKI (*n* (%))	49 (59.8)	47 (67.1)	2 (16.7)	< 0.001
AKI stage 1 (*n* (%))	23 (28)	21 (30)	2 (16.7)	
AKI stage 2 (*n* (%))	4 (4.9)	2 (2.9)	2 (16.7)	
AKI stage 3 (*n* (%))	6 (7.3)	–	6 (50)	

Reported as mean ± standard deviation unless indicated otherwise. *p* value compares patients who survived or did not need RRT to those who reached mortality or required RRT. Adapted from reference 4. *AKI* acute kidney injury, *ISS* injury severity score, *IQR* interquartile range, *RRT* renal replacement therapy, *Mass.* massive

Eighty-four (84) metabolites were identified and quantified in each urine sample. Of these, 11 were identified as drugs or drug metabolites and excluded from further analysis (Additional file 1). The remaining 73 metabolites are listed in Table 2 and their peaks are labeled in Additional file 2.

**Table 2:** List of 73 metabolites used in analysis

Identified metabolites	
1-Methylnicotinamide	Hydroxyacetone
1,6-Anhydro-beta-D-glucose	Hypoxanthine
2-Aminoadipate	Indole-3-acetate
2-Aminobutyrate	Isoleucine
2-Hydroxybutyrate	Lactate
2-Hydroxyisobutyrate	Lactose
2-Hydroxyvalerate	Leucine
2-Oxoglutarate	Lysine
3-Aminoisobutyrate	Malonate
3-Hydroxybutyrate	Methylguanidine
3-Hydroxyisobutyrate	Methylmalonate
3-Hydroxyisovalerate	myo-Inositol
3-Indoxylsulfate	N-Methylhydantoin
3-Methyl-2-oxovalerate	N,N-Dimethylglycine
Acetate	O-Acetylcarnitine
Acetoacetate	O-Phosphocholine
Acetone	Phenylacetylglycine
Alanine	Phenylalanine
Betaine	Pyridoxine
Carnitine	Pyruvate
Choline	Sarcosine
cis-Aconitate	Succinate
Citrate	Tartrate
Creatine	Taurine
Creatine phosphate	Threonine
Creatinine	Trigonelline
Dimethylamine	Trimethylamine
Ethanolamine	Trimethylamine N-oxide
Formate	Tyramine
Fucose	Tyrosine
Fumarate	Urea
Glucose	Valine
Glutamine	Xanthine
Glycine	Xanthosine
Hippurate	pi-Methylhistidine
Histamine	tau-Methylhistidine
Histidine	

List of 73 metabolites used in analysis of outcomes after exclusion of exogenous metabolites (Additional file 1)

### Partial least squares discriminant analysis

The *R*^2^ and *Q*^2^ values and *p* values of the PLS-DA models were evaluated (Table 3). Based on model *R*^2^, *Q*^2^, and permutation *p* values, ISS and AKI diagnosis were excluded from further analysis (Additional files 3 and 4). The mortality/RRT PLS-DA model had an insignificant *p* value, however, we postulated that by relaxing the threshold of *p* < 0.05 we would be able to draw useful conclusions that had clinical significance since mortality and renal replacement therapy are important combat casualty and AKI outcomes [1, 4, 15]. Furthermore, the other model statistics for mortality/RRT were acceptable and comparable to the AKI stage PLS-DA model. Therefore, we proceeded with our analyses of mortality/RRT and AKI stage.

**Table 3:** PLS-DA model values

Outcome	*R*^2^	*Q*^2^	Permutation *p* value
**AKI Stage: 0–1 (none-mild) or 2–3 (moderate-severe)**	0.487	0.302	0.013
**Mortality/RRT: Yes or no**	0.536	0.324	0.173
AKI diagnosis: Yes or no	0.382	0.22	0.306
Injury severity score (ISS): < 25 or ≥ 25	0.46	0.265	0.402

Bolded: acceptable model statistics and included in further analysis. *RRT* renal replacement therapy, *AKI* acute kidney injury

### Mortality and renal replacement therapy

**Fig. 1:**
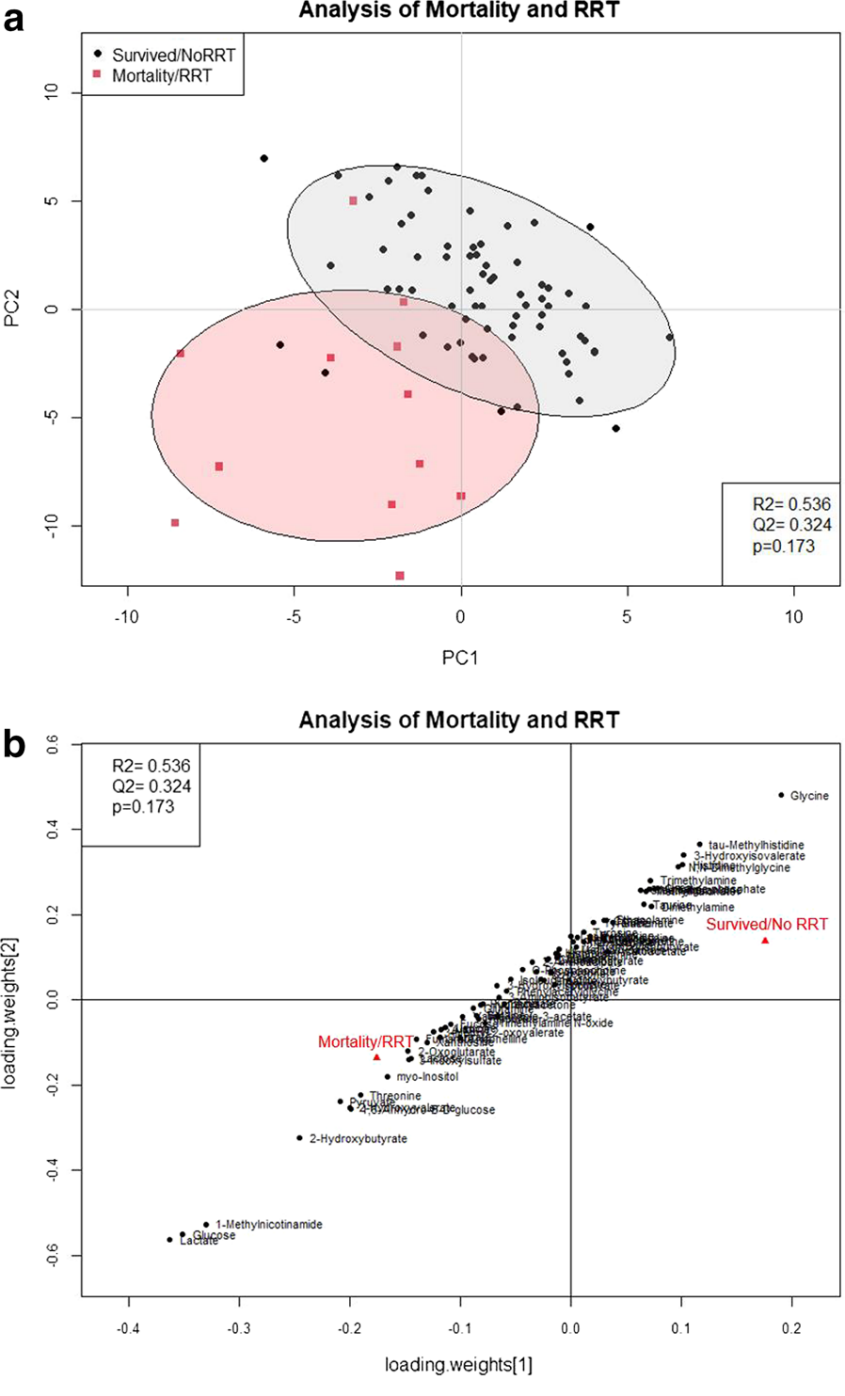
Analysis for mortality and renal replacement therapy. **a** PLS-DA scores plot of urine samples collected from patients who reached the endpoint of mortality or RRT (mortality/RRT, square, *n* = 12) or who survived or did not need RRT (Survived/NoRRT, circle). Two (2) of the patients in the mortality/RRT group did not have or develop AKI. Each circle and square represents a urine sample. The ellipses represent the 95% confidence interval for the groups. **b** Loadings plot for mortality and RRT. Loadings show how metabolites contribute to separation seen in the scores plot. *PC* principal component, *RRT* renal replacement therapy

Twelve (12) patients reached the endpoint of mortality or need for RRT. Two (2) of the patients in the mortality/RRT group did not have or develop AKI. The PLS-DA scores plot (Fig. 1a) shows separation between patients who reached the endpoint versus those who did not. The corresponding PLS-DA loadings plot (Fig. 1b) and VIP scores table (Table 4) show that 9 metabolites were significantly associated with mortality/RRT (*p* < 0.05). In the loadings plot, metabolites trending towards the bottom left were present in higher levels in the mortality/RRT group. Metabolites trending to the top right were present in lower levels in the mortality/RRT group. Metabolites 1-methylnicotinamide, lactate, and glucose were associated the mortality/RRT group while glycine was associated with those who survived or did not need RRT.

**Table 4:** Two-component VIP Scores and *p* values for Mortality and RRT

Metabolite/biomarker	VIP score	*p* value
Lactate	**3.78**	**< 0.001**
Glucose	**3.66**	**< 0.001**
1-Methylnicotinamide	**3.45**	**< 0.001**
2-Hydroxybutyrate	**2.55**	**0.008**
Glycine	**2.37**	**0.01**
Pyruvate	**2.20**	**0.02**
2-Hydroxyvalerate	**2.09**	**0.04**
1,6-Anhydro-ß-D-Glucose	**2.07**	**0.006**
Threonine	**2.00**	**0.005**
Myo-Inositol	**1.76**	0.07

The table includes the metabolites/biomarkers with the top 10 VIP scores (bolded) in addition to metabolites with significant *p* values (*p* < 0.05, bolded) for mortality and RRT. Metabolites are ordered by VIP score. *RRT* renal replacement therapy

### Acute kidney injury stage

**Fig. 2:**
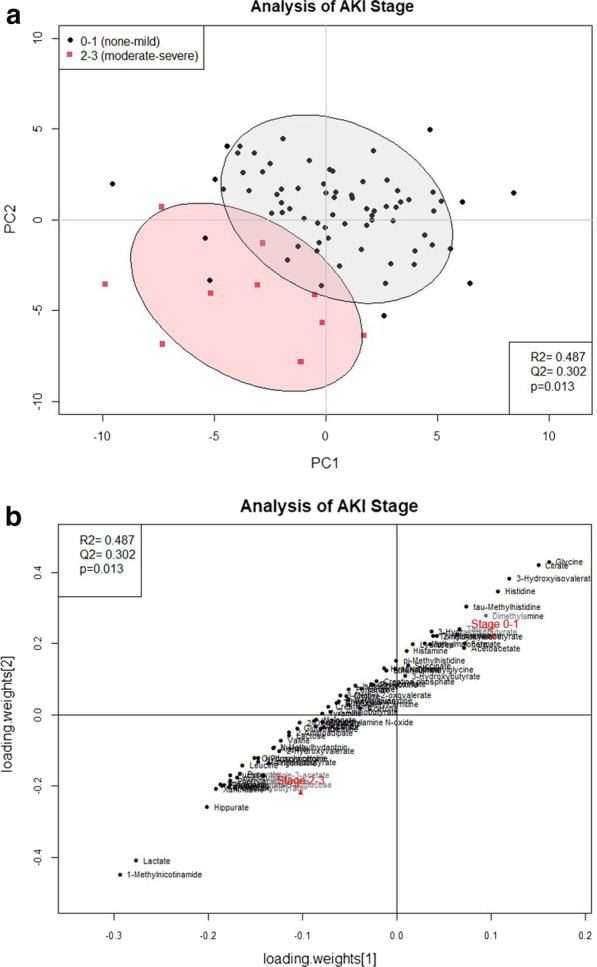
Analysis for acute kidney injury (AKI) stage. **a** PLS-DA scores plot of urine samples collected from patients who were AKI stages 0–1 (none-mild, circle) or stages 2–3 (moderate-severe, square, *n* = 10). Each circle and square represents a urine sample. The ellipses represent the 95% confidence interval for the groups. **b** Loadings plot for AKI stage. Loadings show how metabolites contribute to separation seen in the scores plot. *PC* principal component

Ten (10) patients had moderate to severe AKI (stage 2–3). The majority of these subjects (*n* = 6) had developed AKI prior to the urine sample being taken. The PLS-DA scores plot (Fig. 2a) shows separation between patients who had moderate to severe AKI versus those who had none or mild AKI (stage 0–1). The corresponding PLS-DA loadings plot (Fig. 2b) and VIP scores table (Table 5) show that 11 metabolites were significantly associated with moderate to severe AKI (*p* < 0.05). In the loadings plot, metabolites in the bottom left were present in higher levels in the moderate to severe AKI group. Metabolites in the top right were present in lower levels in the moderate to severe AKI group. Lactate and 1-methylnicotinamide were associated with the moderate to severe AKI group and glycine was associated with the none or mild AKI group.

**Table 5:** Two-component VIP Scores and *p* values for AKI Stage

Metabolite/biomarker	VIP score	*p* value
1-Methylnicotinamide	**2.92**	**< 0.001**
Lactate	**2.73**	**< 0.001**
Glycine	**2.32**	0.06
Citrate	**2.27**	0.09
3-Hydroxyisovalerate	**2.09**	0.13
Hippurate	**1.95**	**0.04**
Histidine	**1.89**	0.3
Xanthosine	**1.86**	0.1
3-Indoxylsulfate	**1.82**	**0.05**
Tartrate	**1.80**	**0.02**
Threonine	1.78	**0.02**
Phenylacetylglycine	1.72	**0.04**
1,6-Anhydro-ß-D-Glucose	1.71	**0.03**
Glucose	1.66	**0.002**
Pyruvate	1.62	**0.05**
Indole-3-Acetate	1.36	**0.05**

The table includes the metabolites/biomarkers with the top 10 VIP scores (bolded) in addition to metabolites with significant *p* values (*p* < 0.05, bolded) for AKI stage. Metabolites are ordered by VIP score

### 1-Methylnicotinamide, lactate, and glycine

**Fig. 3:**
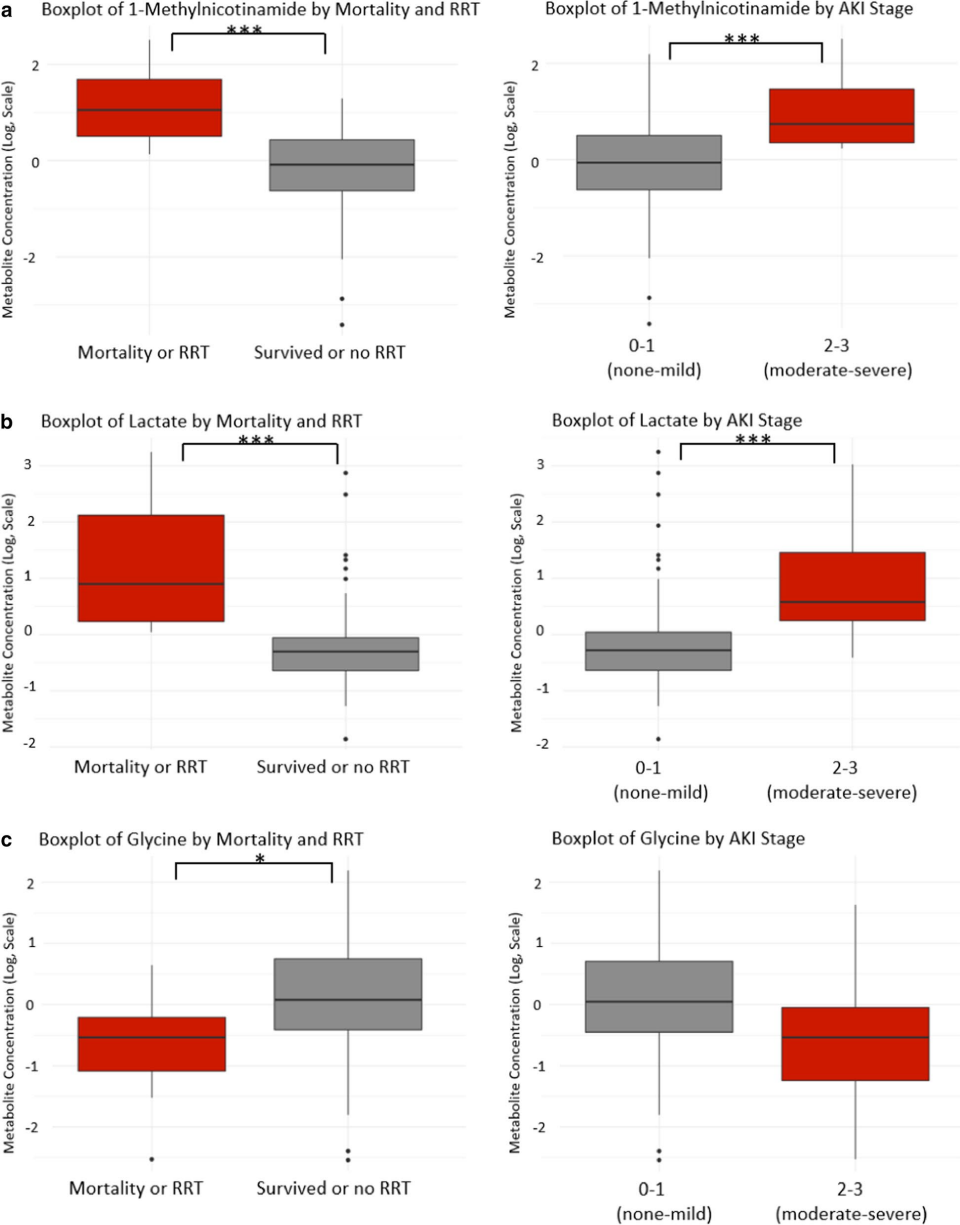
Boxplots of 1-methylnicotinamide, lactate, and glycine and their relationship to outcomes. Metabolite concentrations were normalized by urine output, log-transformed and autoscaled. Boxplots were created using values for the median and interquartile range of each metabolite for each group. **a** Boxplots for 1-methylnicotinamide levels by mortality/RRT and AKI stage. **b** Boxplots for lactate levels by mortality/RRT and AKI stage. **c** Boxplots for glycine levels by mortality/RRT and AKI stage. ***p < 0.001; *p < 0.05; *RRT* renal replacement therapy

Based on the PLS-DA loadings plots, VIP scores, and univariate statistics, 1-methylnicotinamide and lactate were most associated with adverse outcomes while glycine was associated with better outcomes. Lactate and 1-methylnicotinamide levels were significantly higher in patients who had more severe AKI and in patients who reached the endpoint of mortality/RRT versus those who did not (Fig. 3a, b, *p* < 0.001). Glycine levels are significantly lower in mortality/RRT patients versus survived or no RRT patients (Fig. 3c, *p* = 0.01) and lower in patients who had more severe AKI (Fig. 3c, *p* = 0.06). The only metabolite to be significantly associated with all adverse outcomes was 1-methylnicotinamide. In addition to AKI stage and mortality/RRT, 1-methylnicotinamide was significantly (*p* < 0.05) higher in patients with a higher injury severity score (25 ≤) and patients diagnosed with AKI (Additional file 5).

**Fig. 4:**
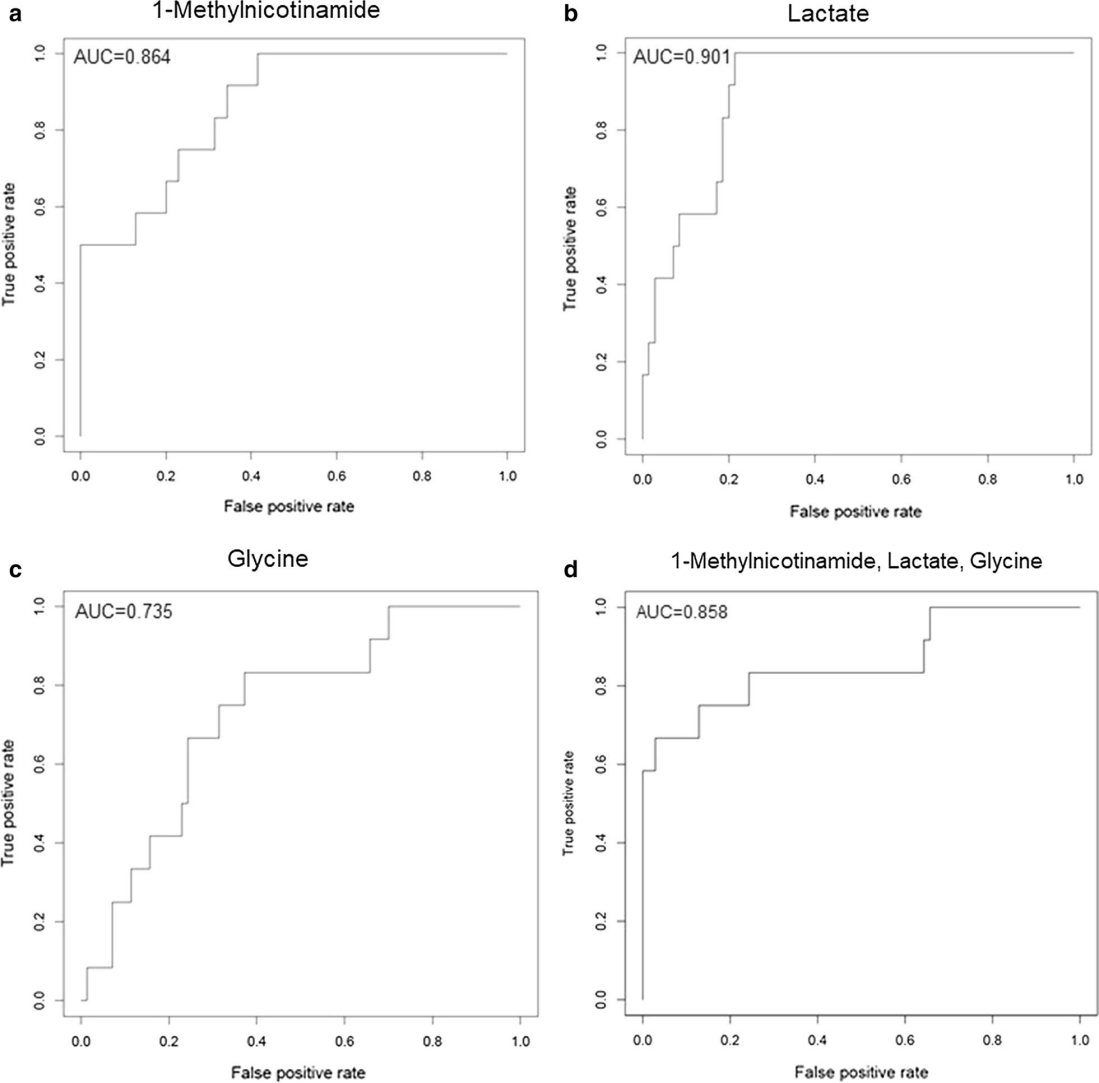
Receiver operating characteristic (ROC) curves for mortality/RRT. **a** 1-methylnicotinamide (AUC = 0.864), **b** lactate (AUC = 0.901), **c** glycine (AUC = 0.735), and **d** all 3 (AUC = 0.858). *AUC* area under the curve

Receiver operating characteristics curves of 1-methylnicotinamide, lactate, glycine, and the three metabolites as a panel were generated to analyze the metabolites’ diagnostic ability for mortality or need for renal replacement therapy (Fig. 4). Based on the area under the curve (AUC), lactate alone had the greatest ability (AUC = 0.901, Fig. 4b), followed by 1-methylnicotinamide (AUC = 0.864, Fig. 4a), then glycine (AUC = 0.735, Fig. 4c). Combined as a panel (Fig. 4d), these biomarkers had a good diagnostic ability for mortality or need for RRT (AUC = 0.858).

## Discussion

In this study, we identified 3 predictive urinary biomarkers of mortality or need for renal replacement therapy in combat casualties: lactate, 1-methylnicotinamide, and glycine. These 3 metabolites are also associated with AKI stage. We investigated the relationships and roles these metabolites have to each other, to combat trauma, and to AKI through literature reviews and pathway identification.

### Metabolites and pathways

**Fig. 5:**
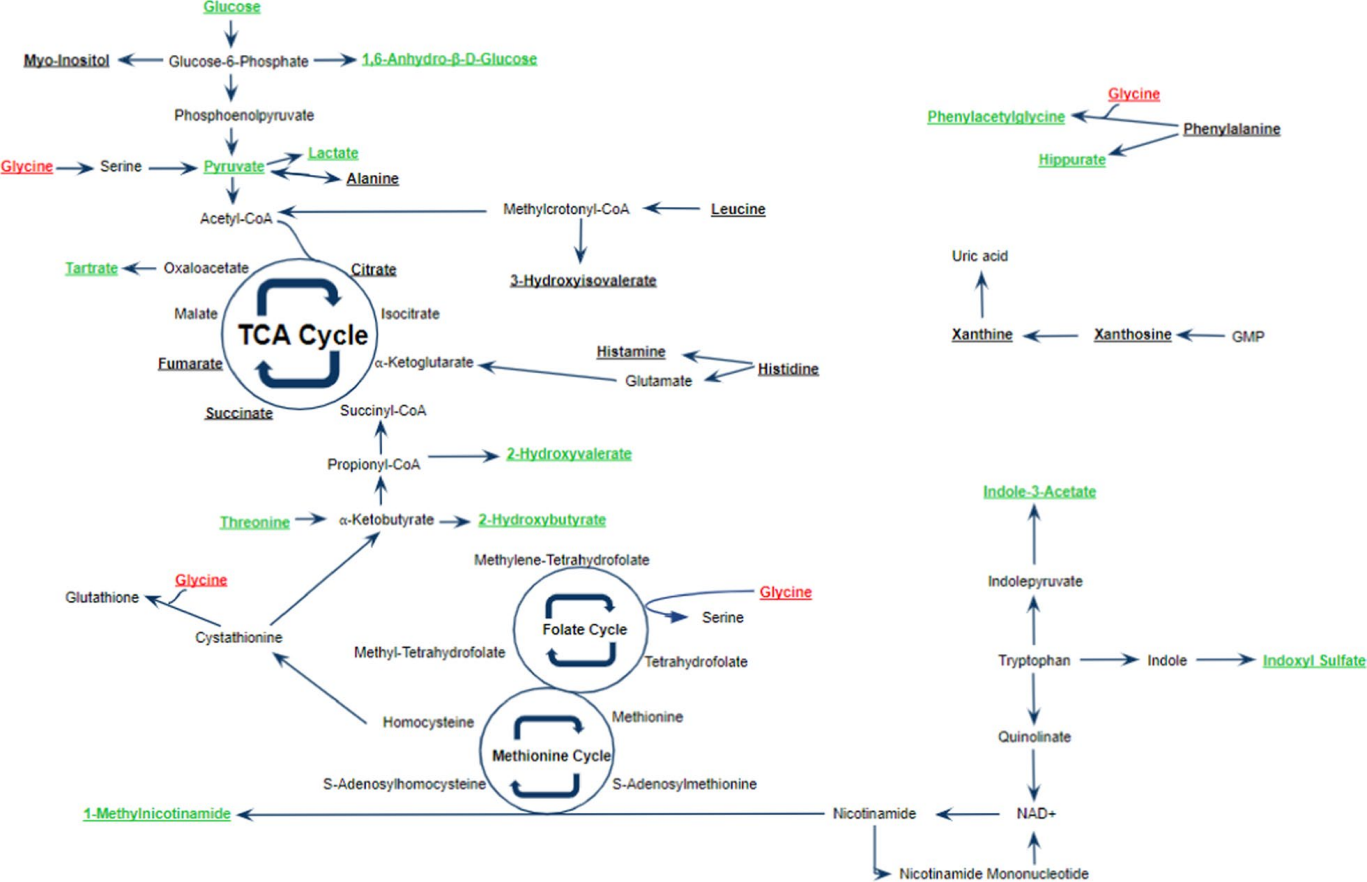
Relationship of various metabolic pathways for AKI and combat injury. Metabolites included in the schematic were either a top 10 VIP metabolite or were statistically significant (*p* < 0.05). Metabolic pathways: glycolysis, TCA cycle, folate and methionine cycles, Cori and Cahill cycles, gut microbe metabolism, and NAD+ salvage pathway. Bolded metabolites were identified in the ^1^H-NMR analysis. Metabolites in green were significantly higher in patients who reached the endpoint or mortality/RRT and/or had more severe AKI. Metabolites in red were significantly lower in patients who reached the endpoint or mortality/RRT and/or had more severe AKI

To identify the pathways involved and to determine the relationships between the metabolites that distinguished between disease severity and mortality/RRT, we referenced the Kyoto Encyclopedia of Genes and Genomes (KEGG) Pathway Database (Kanehisha Laboratories, Japan), Human Metabolome Database (HMDB), The Metabolomics Innovation Center, Canada), and reviewed published literature. Metabolites included in the schematic (Fig. 5) were either a top 10 VIP metabolite or were statistically significant (*p* < 0.05). Based on these results, we propose 5 pathways that may be involved in patient outcomes: gut microbiome metabolism, glycolysis, TCA cycle, the methionine and folate cycles, and the NAD+ salvage pathway (Fig. 5) [16–21]. We offer some potential explanations on why these metabolite levels might be altered.

#### Gut microbiome metabolism

Research of the gut microbiome has expanded exponentially over the recent years, and we are constantly finding new links between the gut microbiome and other organ systems. Although limited, research has been published about the interactions between the intestinal microbiota and kidneys [22–25]. The gut microbiome produces compounds such as indoles (indoxyl sulfate and indole-3-acetate), hippurate, and phenylacetylglycine, which are normally excreted in the urine and non-toxic [20, 23, 24]. However, when there is dysbiosis, or microbial imbalance, these compounds are produced at an increased rate [24]. An accumulation of these uremic toxins in the kidneys can cause inflammation and damage. In our study, the uremic toxins indoxyl sulfate, indole-3-acetate, hippurate, and phenylacetylglycine were associated with the moderate-severe AKI group (Fig. 2, Table 5). These toxins alone might cause kidney disease or exacerbate an existing kidney disease [24, 25]. However, AKI could lead to an accumulation of these toxins as well [24, 25]. Either way, it has been suggested that part of the treatment and prevention for kidney diseases is controlling the dysbiosis through pre/probiotics or microbiome transplants so that fewer toxins are being produced [23].

Dysbiosis in this patient population may have been caused by combat injury. A few studies have shown that traumatic injury causes a significant change in the intestinal microbiome, and this change can happen in less than 72 h. Even though these studies investigated different forms of traumatic injury (burn, traumatic brain injury (TBI), or blunt/penetrating trauma without TBI), they all saw significant increases in aerobic bacteria, such as Enterobacteria and Enterococci species [26–28]. These species were also significantly increased in patients with chronic kidney disease (CKD) and end stage renal disease (ESRD) [23].

#### Glycolysis and TCA cycle

Glucose production is central to the metabolic response to trauma [16]. Glucose is used in glycolysis to produce pyruvate, which is then used for the TCA cycle. In our data, significant (*p* < 0.05) increases in glucose and pyruvate levels were associated with mortality or the need for RRT. Because this group had more severe trauma (Table 1), an upregulated metabolic response would be expected compared to the group who survived or did not need RRT.

The TCA cycle produces products used in oxidative phosphorylation, which makes ATP and requires oxygen. In this study, the patient population suffered severe blood loss and hemorrhagic shock from combat injury, which means there most likely was not enough oxygen to sustain aerobic metabolism. Therefore, anaerobic metabolism is likely upregulated, leading to increased lactate levels [29]. This is supported by Table 1: the group of patients in the mortality/RRT group had a significantly higher percentage of patients receive massive transfusions (*p* = 0.006), had significantly higher average injury severity scores (*p* = 0.002), and significantly higher median serum lactate levels (*p* < 0.001). Additionally, lactate levels were significantly higher in patients who had adverse outcomes (Fig. 3b).

**Fig. 6:**
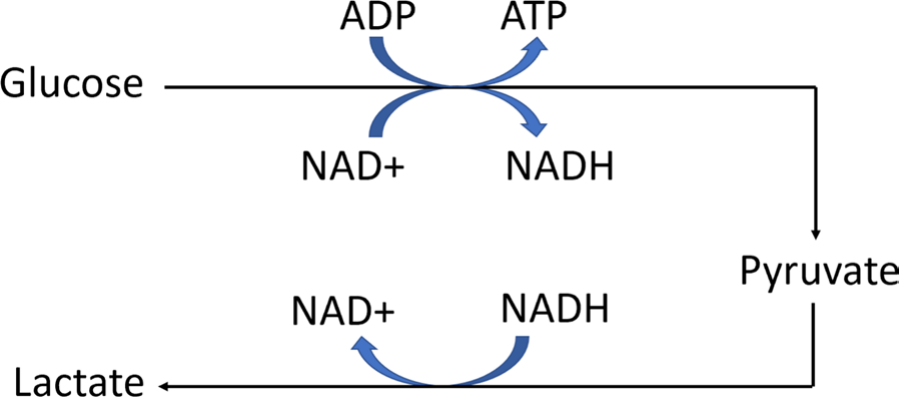
Simplified pathway of lactic acid fermentation. This anaerobic metabolism occurs when there is not enough oxygen to run the TCA cycle and oxidative phosphorylation. Glycolysis converts glucose to pyruvate, producing ATP and NADH. Pyruvate makes lactate instead of going into the TCA cycle. This reaction produces NAD+ from NADH. Adapted from Reference [30]

During anaerobic production of ATP, glucose is still converted to pyruvate via glycolysis, which converts NAD+ into NADH. However, instead of proceeding to the TCA cycle, pyruvate is used to make lactate (Fig. 6). The conversion of pyruvate to lactate uses NADH to make NAD+, which can be used in glycolysis. This process, lactic acid fermentation, is crucial to sustain the NAD+ pool that is used for glycolysis in times of limited oxygen supply [30].

#### Methionine and folate cycles

Mortality/RRT patients had significantly lower levels of glycine (*p* < 0.05) and significantly higher levels of 2-hydroxybutyrate (*p* < 0.05) compared to patients who survived or did not need RRT (glycine Fig. 3c-2-hydroxybutyrate Additional file 6). These findings are similar in other studies of traumatic injury and suggests that glycine and 2-hydroxybutyrate are biomarkers of oxidative stress. In a study of traumatic brain injury, Dash et al. discovered that patients with severe and mild TBI had significantly increased levels of 2-hydroxybutyrate and significantly decreased levels of glycine in their plasma compared to heathy volunteers’ plasma [31]. In a study of burn injury using a porcine model, Hendrickson et al. reported that 2-hydroxybutyrate was significantly increased and glycine was significantly decreased over a 72 h period after burn injury [7]. In a rat model of ischemic acute kidney injury, Fox et al. found that glycine was significantly reduced in the AKI rats 24 h after injury compared to baseline and when comparing the sham rats to the AKI rats at 24 h [32]. Although these data were from plasma and our analysis was done in urine, the findings for 2-hydroxybutyrate and glycine were similar.

Glycine is used in the production of glutathione, a key antioxidant produced from the methionine cycle that eliminates reactive oxygen species (Fig. 5) [18]. In times of severe oxidative stress as with combat injury, upregulation of glutathione production has been shown to occur at a faster rate than the synthesis of glycine [18, 32]. This might explain why glycine levels are significantly lower in the mortality/RRT patients (Fig. 3c). Of note, glutathione was not identified in our urine samples. Upregulation of α-ketobutyrate production occurs during times of oxidative stress as well, and 2-hydroxybutyrate is a by-product of α-ketobutyrate production (Fig. 5) [19]. Thus, the increased production of α-ketobutyrate may cause an accumulation of 2-hydroxybutyrate.

Another relevant reaction of the methionine cycle is the conversion of S-adenosylmethionine (SAM) to S-adenosylhomocysteine (SAH) using the enzyme nicotinamide N-methyltransferase (NNMT) (Fig. 5) [18]. This reaction is involved in another pathway: the NAD+ salvage pathway.

#### NAD+ salvage pathway

**Fig. 7:**
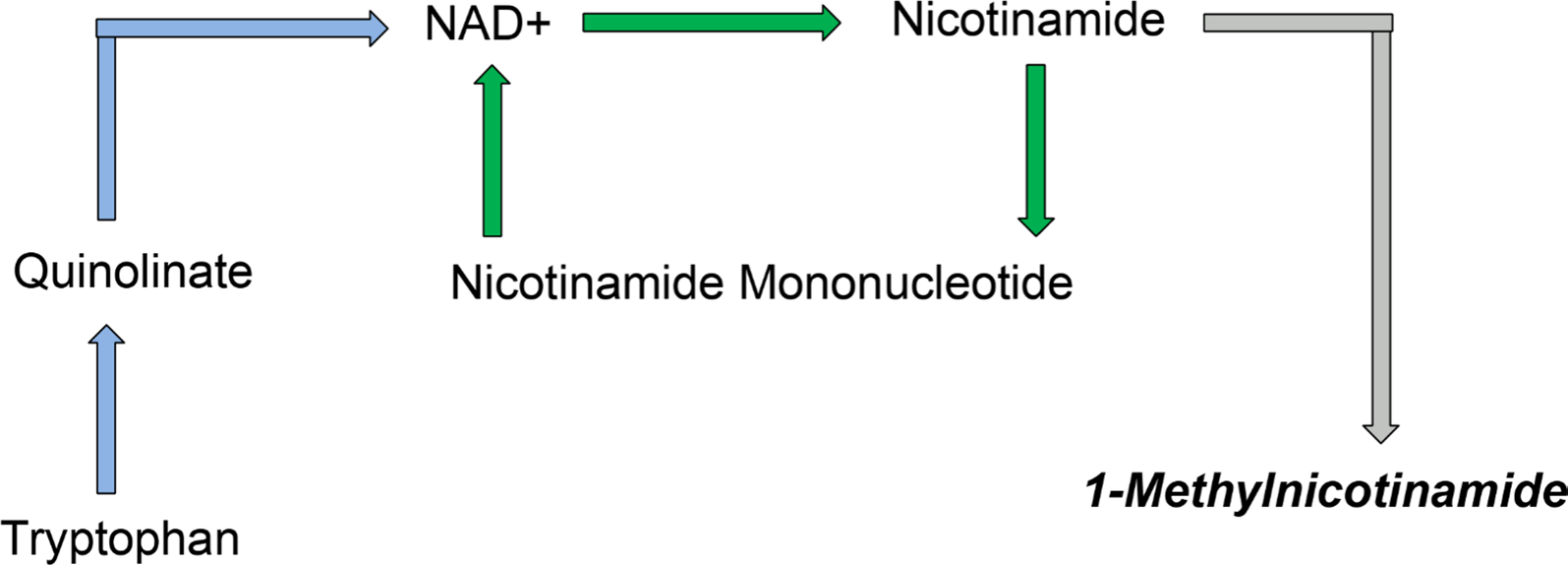
Simplified de novo NAD+ and NAD+ salvage pathways. De novo pathway (blue arrows) makes NAD+ from tryptophan and quinolinate. The salvage pathway (green arrows) makes NAD+ from nicotinamide and nicotinamide mononucleotide. 1-methylnicotinamide is a by-product of the salvage pathway. Adapted from reference [21]

NAD+ is used in the TCA cycle to make NADH for oxidative phosphorylation and when glycolysis is upregulated during times of oxidative stress to make ATP. NAD+ is also used by sirtuins. Sirtuins regulate energy metabolism and are NAD+-dependent enzymes that are involved in many pathways such as lipid metabolism, oxidative stress, urea cycle, TCA cycle, and amino acid metabolism [33]. Two pathways can synthesize NAD+: de novo synthesis and the salvage pathway (Fig. 7).

The de novo pathway synthesizes NAD+ from tryptophan while the salvage pathway uses Vitamin B_3_ compounds such as nicotinamide to make NAD+. Until recently, the de novo pathway was thought to be a minor contributor to intracellular NAD+ levels. However, in a recent study, Poyan Mehr et al. suggests that the de novo pathway plays a bigger role in NAD+ production and AKI risk [21]. The authors showed that in ischemic AKI, levels of the enzyme quinolinate phosphoribosyltransferase (QPRT) were reduced. This enzyme is the last step in the de novo pathway, converting quinolinate into NAD+. The downregulation of this enzyme caused significant decreases in renal NAD+ levels and an increased risk for adverse outcomes [21]. However, there may have been upregulation of the salvage pathway to replenish the NAD+ levels.

The kidneys readily convert nicotinamide into NAD+ via the salvage pathway which tightly regulates nicotinamide levels, keeping them low. The conversion of nicotinamide to nicotinamide mononucleotide produces the by-product 1-methylnicotinamide, which is transported out of the cell and excreted in the urine (Fig. 7) [33]. Nicotinamide N-methyltransferase (NNMT), the enzyme that converts SAM to SAH, is used to make 1-methylnicotinamide from nicotinamide (Fig. 5) [34]. As discussed earlier, the methionine cycle is upregulated during times of oxidative stress to make more glutathione. This means increased NNMT activity to make SAM into SAH. During AKI, if the de novo pathway is downregulated then there may be upregulation of the salvage pathway to sustain NAD+ levels. This would mean more nicotinamide is being used to make NAD+, which means more 1-methylnicotinamide is being produced by NNMT as well. The use of NNMT in the methionine cycle (oxidative stress) and salvage pathway (ischemic AKI) might explain why 1-methylnicotinamide was significantly increased in the mortality/RRT patients and patients who had moderate-severe AKI.

### 1-Methylnicotinamide, lactate, and glycine as biomarkers of AKI severity and predictive markers of mortality and renal replacement therapy

Of the 73 metabolites identified in this study, we identified 1-methylnicotinamide, lactate, and glycine as predictive biomarkers for mortality or need for RRT at time of admission and as biomarkers associated with AKI severity. When comparing the ROC curves for the metabolites, it was not surprising that lactate had a high AUC (Fig. 4b, AUC = 0.901) because lactate levels are strongly associated with trauma severity [27, 35, 36]. The mortality/RRT group had more severe trauma indicated by significantly higher median serum lactate levels, median injury severity scores, and percentage of patients requiring a massive transfusion (Table 1). Therefore, lactate is a good predictor for mortality or need for RRT, and is not specific to AKI.

1-methylnicotinamide is perhaps the most promising novel biomarker (Fig. 4a, AUC = 0.864). It is significantly (*p* < 0.05) elevated in mortality/RRT patients, in those with more severe disease, and in those with more severe injuries (Fig. 3a, Additional file 5B). More work must be done to elucidate the relationships between 1-methynicotinamide, the methionine cycle, oxidative stress, and poor outcomes in AKI and critically ill patients.

The AUC for glycine was not as high as lactate and 1-methylnicotinamide, however, it bears consideration as a biomarker that is decreased in patients with more severe disease and worse outcomes (Fig. 4c, AUC = 0.735). Glycine was significantly decreased in the mortality/RRT group, and may be a useful biomarker of oxidative stress in traumatic injury that can predict adverse outcomes and is not unique to AKI [7, 31, 32].

We evaluated a panel of these metabolic biomarkers (lactate, 1-methylnicotinaide, and glycine) and found that it is predictive for mortality and RRT (Fig. 4d, AUC = 0.858). In addition to these 3 metabolites, Stewart et al. identified protein biomarkers that were significantly associated with mortality and RRT as well [1]. The biomarkers identified in this study and in the Stewart et al. study may be used to enrich prediction models for mortality and renal replacement therapy.

### Limitations and future directions

This observational pilot study using data analyzed in a retrospective fashion had several limitations. We were limited to patients who likely developed AKI from a single condition (trauma/combat injury), although AKI can be caused by a variety of other conditions such as medications and heart disease [4]. Of the patients diagnosed with AKI, roughly 50% had been diagnosed with AKI prior to the urine draw. Sixty percent of the patients in the moderate-severe AKI group had been diagnosed with AKI prior to the urine draw. Therefore, we could not determine the diagnostic or prognostic ability of these biomarkers since urine draws did not exclusively occur before or after an AKI diagnosis. Additionally, the study population was almost 100% male and most patients were under the age of 40. This does not allow us to evaluate predictive biomarkers based on age or sex. The study population was small, which limited the statistical power of the study and required the use of groups for the outcomes, such as mortality/RRT and grouping AKI stages into moderate-severe and none-mild. Due to the exploratory nature of this study, multiple comparisons testing was not performed. This study did not have any long term follow up and not all data was collected prospectively [1].

For future studies, there should be a more diverse patient population that encompasses multiple suspected causes of AKI and different patient age and sex. Future studies should validate 1-methylnicotnamide as a prognostic biomarker of mortality and renal replacement therapy, either through targeted or untargeted analysis. A subsequent study should further investigate the prognostic or diagnostic ability of the metabolites identified in this study for AKI stage. In a related note, AKI diagnosis did not have strong model statistics, but AKI severity did. This could indicate that mild AKI has similar characteristics and outcomes to patients who do not have AKI, and moderate-severe AKI patients have similar characteristics and outcomes. This may warrant further investigation. Larger studies are necessary to determine how these metabolites can be utilized in risk prediction models such as a study that performs a targeted analysis for 1-methylnicotinamide, lactate, and glycine in a population of surgical patients or civilian trauma patients.

## Conclusion

In this study, we used proton nuclear magnetic resonance (^1^H-NMR) spectroscopy to identify urinary metabolic biomarkers associated with AKI stage or mortality or need for renal replacement therapy. PLS-DA scores and loadings plots of urine metabolite samples showed separation between the groups for the outcomes of AKI stage and mortality/RRT. Several pathways appeared to be involved in the metabolic response to AKI, including gut microbiome metabolism, glycolysis, TCA cycle, the methionine and folate cycles, and the NAD+ salvage pathway. Three metabolites, lactate, 1-methylnicotinamide, and glycine, were identified as potential predictive biomarkers of mortality and RRT, and their association to AKI severity was evaluated. Moreover, this study suggests that 1-methylnicotinamide is a novel biomarker associated with adverse AKI outcomes. Although this study has provided intriguing results, larger studies are necessary to determine how these metabolites can be utilized in risk prediction models.

## Supplementary Information

**Additional file 1**. Table of excluded metabolites. Metabolites were excluded if they were a drug, from drug metabolism, or other exogenous metabolite.

**Additional file 2**. Labeled NMR spectrum of patient 45 using Chenomx. Includes the 73 identified metabolites listed in Table 2. Concentration values obtained relative to the internal standard TSP. X-axis is chemical shift in parts per million (ppm). Peaks are marked by metabolite number: 1) 1-methylnicotinamide, 2) 1,6-Anhydro-beta-D-glucose, 3) 2-aminoadipate, 4) 2-aminobutyrate, 5) 2-hydroxybutyrate, 6) 2-hydroxyisobutyrate, 7) 2-hydroxyvalerate, 8) 2-oxoglutarate, 9) 3-aminoisobutyrate, 10) 3-hydroxybutyrate, 11) 3-hydroxyisobutyrate, 12) 3-hydroxyisovalerate, 13) 3-indoxylsulfate, 14) 3-methyl-2-oxovalerate, 15) acetate, 16) acetoacetate, 17) acetone, 18) alanine, 19) betaine, 20) carnitine, 21) choline, 22) cis-aconitate, 23) citrate, 24) creatine, 25) creatine phosphate, 26) creatinine, 27) dimethylamine, 28) ethanolamine, 29) formate, 30) fucose, 31) fumarate, 32) glucose, 33) glutamine, 34) glycine, 35) hippurate, 36) histamine, 37) histidine, 38) histamine, 39) hypoxanthine, 40) indole-3-acetate, 41) isoleucine, 42) lactate, 43) lactose, 44) leucine, 45) lysine, 46) malonate, 47) methylguanidine, 48) methylmalonate, 49) myo-inositol, 50) n-methylhydantoin, 51) n,n-dimethylglycine, 52) o-acetylcarnitine, 53) o-phosphocholine, 54) phenylacetylglycine, 55) phenylalanine, 56) pyridoxine, 57) pyruvate, 58) sarcosine, 59) succinate, 60) tartrate, 61) taurine, 62) threonine, 63) trigonelline, 64) trimethylamine, 65) trimethylamine n-oxide, 66) tyramine, 67) tyrosine, 68) urea, 69) valine, 70) xanthine, 71) xanthosine, 72) pi-methylhistidine, 73) tau-methylhistidine.

**Additional file 3**. Analysis for injury severity score (ISS). A) PLS-DA scores plot of urine samples collected from patients who had injury severity scores less than 25 (<25, circle) or greater than or equal to 25 (25≤, square). Each circle and square represents a urine sample. The ellipses represent the 95% confidence interval for the groups. B) Loadings plot for ISS. <25 = patients with an ISS less than 25, 25≤ = patients with an ISS greater than or equal to 25. Loadings show how metabolites contribute to separation seen in the scores plot.

**Additional file 4**. Analysis for acute kidney injury (AKI) diagnosis. A) PLS-DA scores plot of urine samples collected from patients who were diagnosed with AKI (yes, square) or not diagnoses with AKI (no, circle). Each circle and square represents a urine sample. The ellipses represent the 95% confidence interval for the groups. B) Loadings plot for AKI diagnosis. Loadings show how metabolites contribute to separation seen in the scores plot.

**Additional file 5**. Boxplots of 1-methylnicotinamide and the relationship to ISS and AKI diagnosis. Metabolite concentrations were normalized by urine output and log-transformed and autoscaled. Boxplots were created using values for the median and interquartile range of each metabolite for each group. A) Boxplot shows 1-methylnicotinamide levels are significantly higher in patients who were diagnosed with AKI compared to those who were not diagnosed with AKI. B) Boxplot shows 1-methylnicotinamide levels are significantly higher in patients who have higher injury severity scores (25≤) versus those with lower injury severity scores (<25). ISS = injury severity score; AKI = acute kidney injury. * = p<0.05.

**Additional file 6**. Boxplot of 2-hydroxybutyrate and the relationship to mortality/RRT. Boxplots were created using values for the median and interquartile range of each metabolite for each group. Metabolite concentrations were normalized by urine output, log-transformed and autoscaled. Boxplot shows 2-hydroxybutyrate levels are significantly higher in mortality/RRT patients versus patients who survived or did not need RRT. RRT = renal replacement therapy. * = p<0.05.

## Data Availability

The datasets analyzed during the current study are available from the corresponding author on reasonable request.

## Abbreviations

^1^H-NMR: Proton nuclear magnetic resonance
AKI: Acute kidney injury
AUC: Area under the curve
CKD: Chronic kidney disease
ESRD: End stage renal disease
HMDB: Human Metabolome Database
ICU: Intensive care unit
ISS: Injury severity score
KDIGO: Kidney Disease: Improving Global Outcomes
KEGG: Kyoto Encyclopedia of Genes and Genomes
NNMT: Nicotinamide N-methyltransferase
NOESY: Nuclear Overhauser Effect Spectroscopy
PLS-DA: Partial least squares discriminant analysis
QPRT: Quinolinate phosphoribosyltransferase
ROC: Receiver operating characteristics
RRT: Renal replacement therapy
SAH: S-adenosylhomocysteine
SAM: S-adenosylmethionine
TBI: Traumatic brain injury
TSP: 3-(Trimethylsilyl) propionic acid
UO: Urine output
VIP: Variable importance projection
