# Supplementary material for: Urinary metabolites predict mortality or need for renal replacement therapy after combat injury
Source: Crit Care. 2021 Mar 23;25:119. doi: 10.1186/s13054-021-03544-2 (PMC7988986; doi:10.1186/s13054-021-03544-2)
Supplement: Supplementary file 1 — Additional file 1. Table of excluded metabolites. Metabolites were excluded if they were a drug, from drug metabolism, or other exogenous metabolite. [file 13054_2021_3544_MOESM1_ESM.docx]

| **Excluded Metabolites** |
| --- |
| 4-Aminohippurate |
| Acetaminophen |
| Caffeine |
| Dimethyl sulfone |
| Ethanol |
| Gluconate |
| Glycerol |
| Isopropanol |
| Mannitol |
| Methanol |
| Propylene glycol |

Additional File 1. Table of excluded metabolites. Metabolites were excluded if they were a drug, from drug metabolism, or other exogenous metabolite.
