# Supplementary material for: Urinary metabolites predict mortality or need for renal replacement therapy after combat injury
Source: Crit Care. 2021 Mar 23;25:119. doi: 10.1186/s13054-021-03544-2 (PMC7988986; doi:10.1186/s13054-021-03544-2)
Supplement: Supplementary file 2 — Additional file 2. Labeled NMR spectrum of patient 45 using Chenomx. Includes the 73 identified metabolites listed in Table 2. Concentration values obtained relative to the internal standard TSP. X-axis is chemical shift in parts per million (ppm). Peaks are marked by metabolite number: 1) 1-methylnicotinamide, 2) 1,6-Anhydro-beta-D-glucose, 3) 2-aminoadipate, 4) 2-aminobutyrate, 5) 2-hydroxybutyrate, 6) 2-hydroxyisobutyrate, 7) 2-hydroxyvalerate, 8) 2-oxoglutarate, 9) 3-aminoisobutyrate, 10) 3-hydroxybutyrate, 11) 3-hydroxyisobutyrate, 12) 3-hydroxyisovalerate, 13) 3-indoxylsulfate, 14) 3-methyl-2-oxovalerate, 15) acetate, 16) acetoacetate, 17) acetone, 18) alanine, 19) betaine, 20) carnitine, 21) choline, 22) cis-aconitate, 23) citrate, 24) creatine, 25) creatine phosphate, 26) creatinine, 27) dimethylamine, 28) ethanolamine, 29) formate, 30) fucose, 31) fumarate, 32) glucose, 33) glutamine, 34) glycine, 35) hippurate, 36) histamine, 37) histidine, 38) histamine, 39) hypoxanthine, 40) indole-3-acetate, 41) isoleucine, 42) lactate, 43) lactose, 44) leucine, 45) lysine, 46) malonate, 47) methylguanidine, 48) methylmalonate, 49) myo-inositol, 50) n-methylhydantoin, 51) n,n-dimethylglycine, 52) o-acetylcarnitine, 53) o-phosphocholine, 54) phenylacetylglycine, 55) phenylalanine, 56) pyridoxine, 57) pyruvate, 58) sarcosine, 59) succinate, 60) tartrate, 61) taurine, 62) threonine, 63) trigonelline, 64) trimethylamine, 65) trimethylamine n-oxide, 66) tyramine, 67) tyrosine, 68) urea, 69) valine, 70) xanthine, 71) xanthosine, 72) pi-methylhistidine, 73) tau-methylhistidine. [file 13054_2021_3544_MOESM2_ESM.pdf]

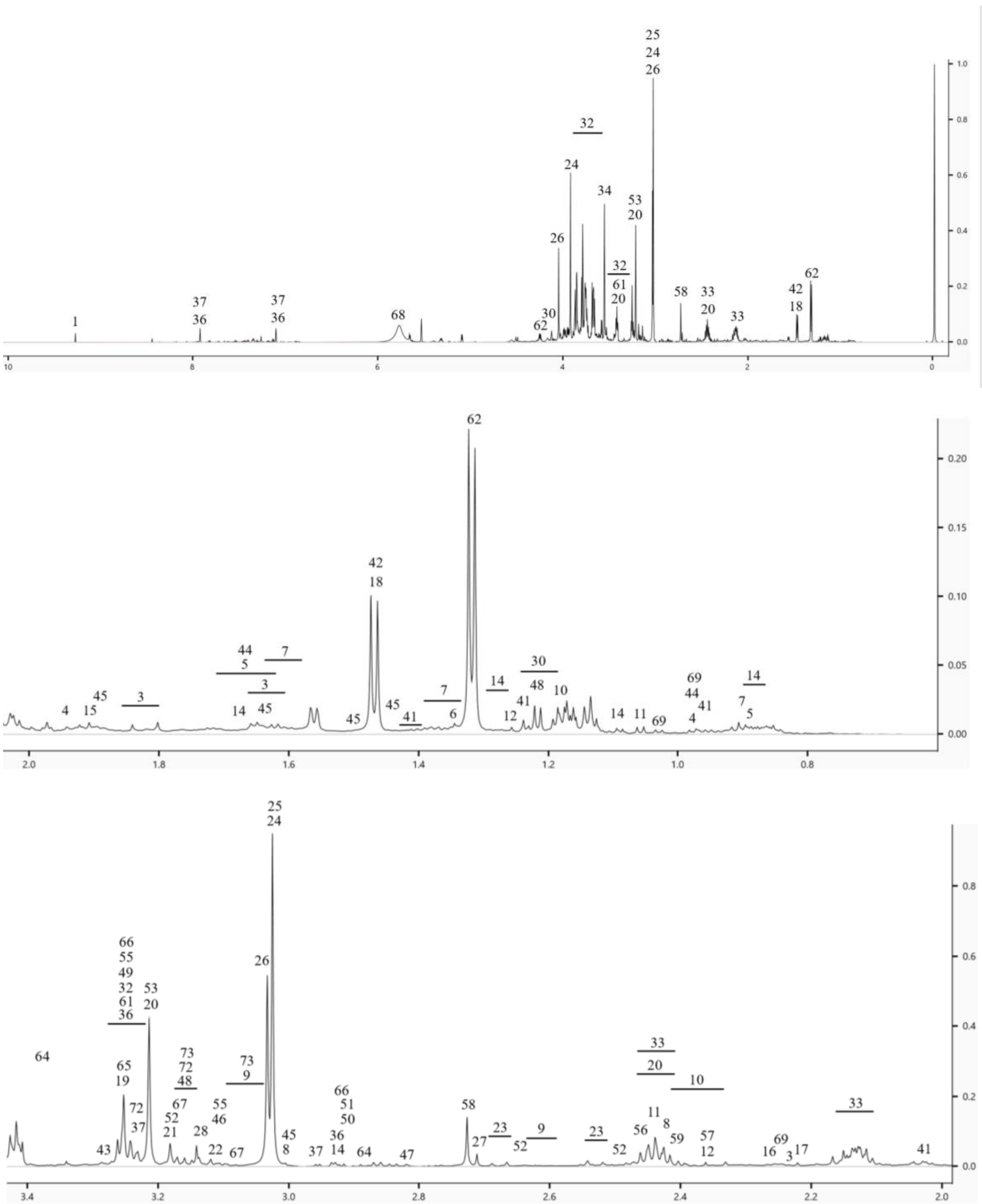

25
24
26
32
24
34
53
20
26
32
61
20
30
62
58
33
20
33
42
18
62
1
37
36
37
36
68
10
8
6
4
2
0
1.0
0.8
0.6
0.4
0.2
0.0
62
42
18
44
5
7
3
45
3
4
15
14
45
45
45
41
7
6
14
12
41
30
48
10
14
11
69
69
44
4
41
7
14
5
2.0
1.8
1.6
1.4
1.2
1.0
0.8
0.20
0.15
0.10
0.05
0.00
25
24
26
66
55
49
32
61
36
53
20
64
65
19
73
72
48
73
9
72
37
67
52
21
28
55
46
43
22
67
45
8
66
51
50
37
36
14
64
47
58
27
23
52
9
23
52
33
20
10
56
11
8
59
57
12
33
16
69
3
17
41
3.4
3.2
3.0
2.8
2.6
2.4
2.2
2.0
0.8
0.6
0.4
0.2
0.0

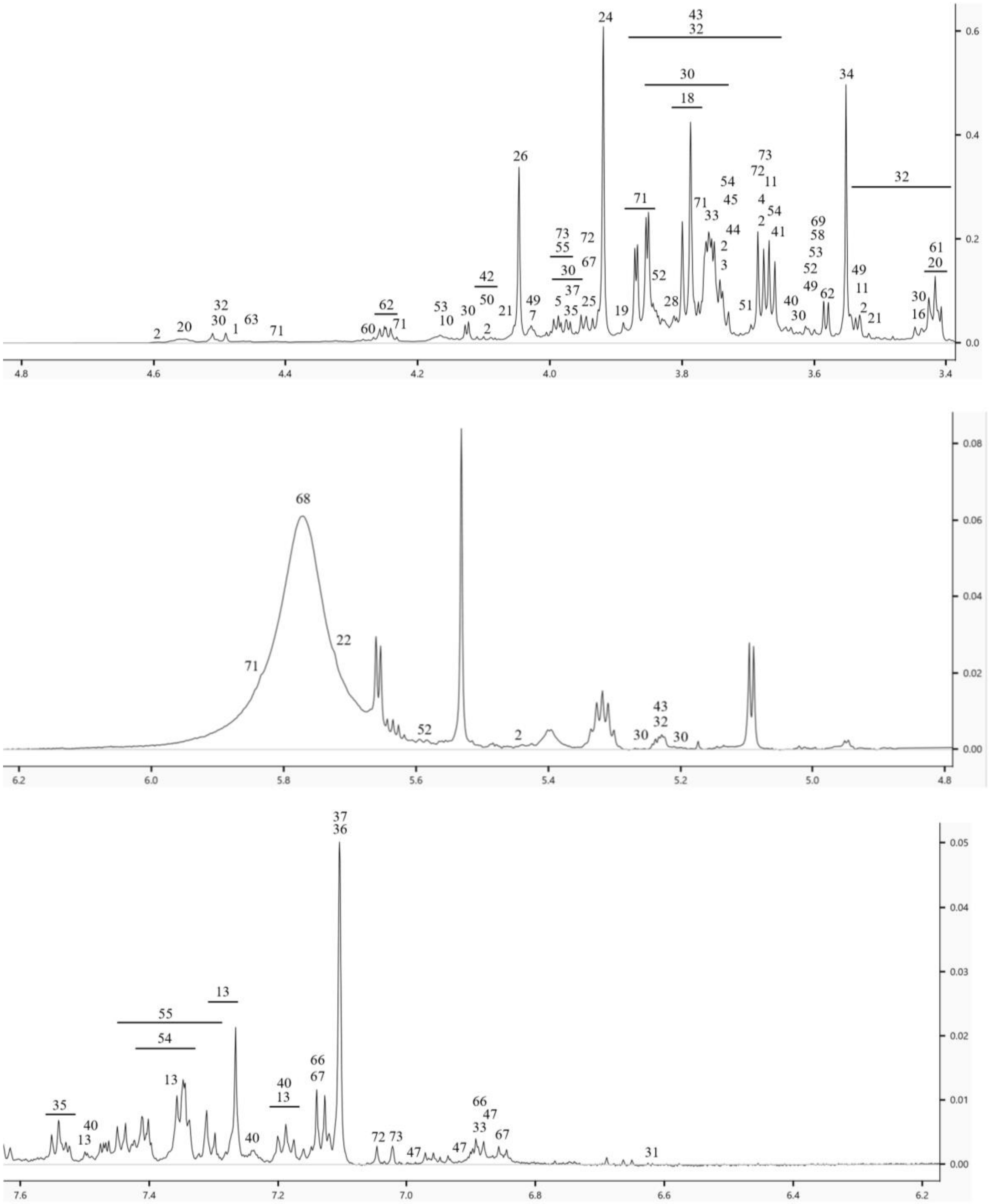

24
43
32
30
18
34
26
71
73
72
11
4
2
54
41
54
45
71
33
44
2
3
52
28
51
69
58
53
52
49
62
40
30
32
49
11
2
21
61
20
30
16
73
55
30
37
5
35
72
67
25
19
42
50
2
21
49
7
53
10
30
62
60
71
2
20
32
30
1
63
71
0.6
0.4
0.2
0.0
4.8
4.6
4.4
4.2
4.0
3.8
3.6
3.4
68
71
22
52
2
43
32
30
30
0.08
0.06
0.04
0.02
0.00
6.2
6.0
5.8
5.6
5.4
5.2
5.0
4.8
37
36
13
55
54
13
35
40
13
40
40
13
66
67
72
73
47
47
66
47
33
67
31
0.05
0.04
0.03
0.02
0.01
0.00
7.6
7.4
7.2
7.0
6.8
6.6
6.4
6.2

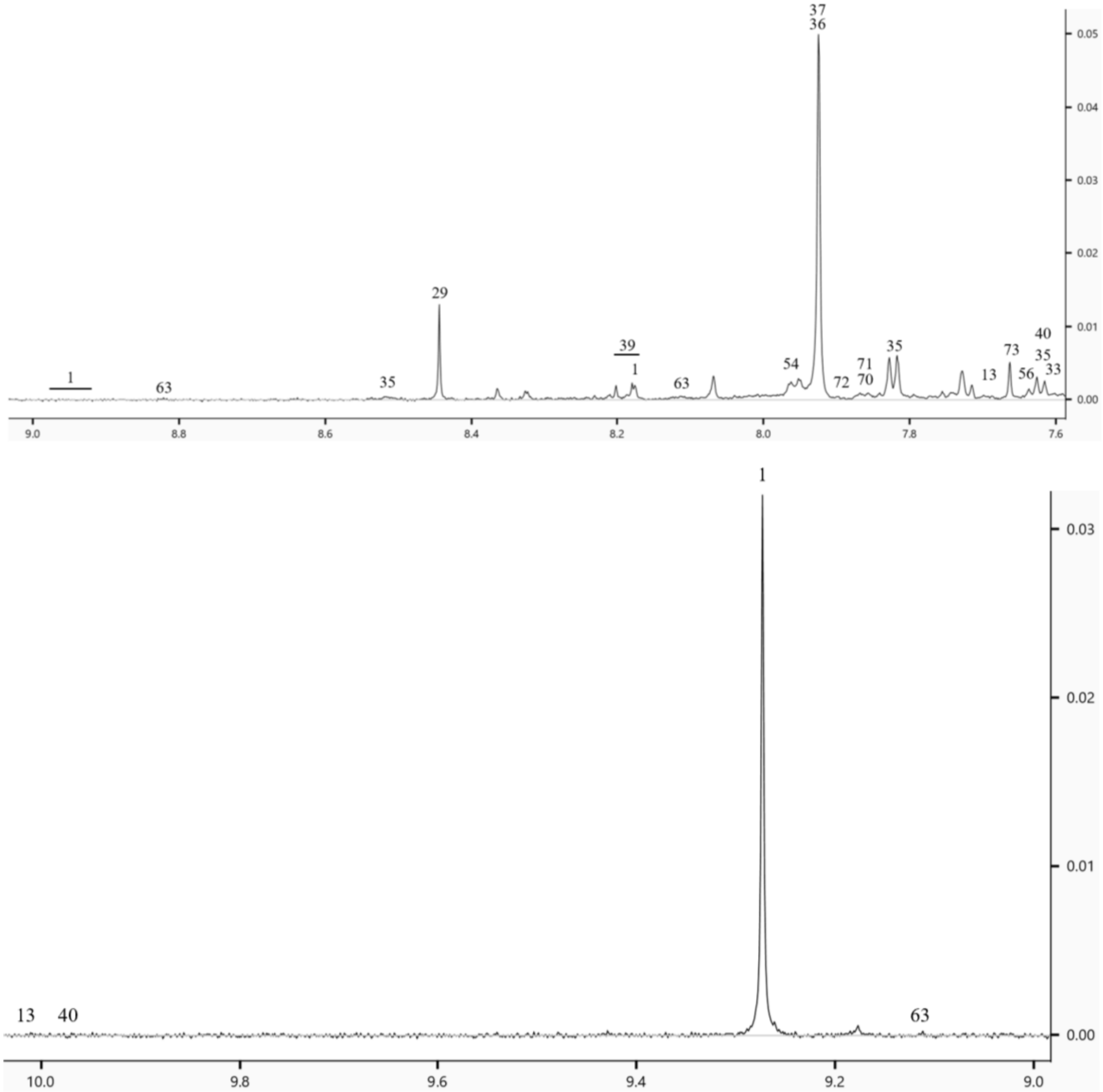

Additional File 2. Labeled NMR spectrum of patient 45 using Chenomx. Includes the 73 identified metabolites listed in Table 2. Concentration values obtained relative to the internal standard TSP. X-axis is chemical shift in parts per million (ppm). Peaks are marked by metabolite number: 1) 1-methylnicotinamide, 2) 1,6-Anhydro-beta-D-glucose, 3) 2-aminoadipate, 4) 2-aminobutyrate, 5) 2-hydroxybutyrate, 6) 2-hydroxyisobutyrate, 7) 2-hydroxyvalerate, 8) 2-oxoglutarate, 9) 3-aminoisobutyrate, 10) 3-hydroxybutyrate, 11) 3-hydroxyisobutyrate, 12) 3-hydroxyisovalerate, 13) 3-indoxylsulfate, 14) 3-methyl-2-oxovalerate, 15) acetate, 16) acetoacetate, 17) acetone, 18) alanine, 19) betaine, 20) carnitine, 21) choline, 22) cis-aconitate, 23) citrate, 24) creatine, 25) creatine phosphate, 26) creatinine, 27) dimethylamine, 28) ethanolamine, 29) formate, 30) fucose, 31) fumarate, 32) glucose, 33) glutamine, 34) glycine, 35) hippurate, 36) histamine, 37) histidine, 38) histamine, 39) hypoxanthine, 40) indole-3-acetate, 41) isoleucine, 42) lactate, 43) lactose, 44) leucine, 45) lysine, 46) malonate, 47) methylguanidine, 48) methylmalonate, 49) myo-inositol, 50) n-methylhydantoin, 51) n,n-dimethylglycine, 52) o-acetylcarnitine, 53) o-phosphocholine, 54) phenylacetylglycine, 55) phenylalanine, 56) pyridoxine, 57) pyruvate, 58) sarcosine, 59) succinate, 60) tartrate, 61) taurine, 62) threonine, 63) trigonelline, 64) trimethylamine, 65) trimethylamine n-oxide, 66) tyramine, 67) tyrosine, 68) urea, 69) valine, 70) xanthine, 71) xanthosine, 72) pi-methylhistidine, 73) tau-methylhistidine.
