# Supplementary material for: Urinary metabolites predict mortality or need for renal replacement therapy after combat injury
Source: Crit Care. 2021 Mar 23;25:119. doi: 10.1186/s13054-021-03544-2 (PMC7988986; doi:10.1186/s13054-021-03544-2)
Supplement: Supplementary file 3 — Additional file 3. Analysis for injury severity score (ISS). A) PLS-DA scores plot of urine samples collected from patients who had injury severity scores less than 25 (<25, circle) or greater than or equal to 25 (25≤, square). Each circle and square represents a urine sample. The ellipses represent the 95% confidence interval for the groups. B) Loadings plot for ISS. <25 = patients with an ISS less than 25, 25≤ = patients with an ISS greater than or equal to 25. Loadings show how metabolites contribute to separation seen in the scores plot. [file 13054_2021_3544_MOESM3_ESM.pdf]

A

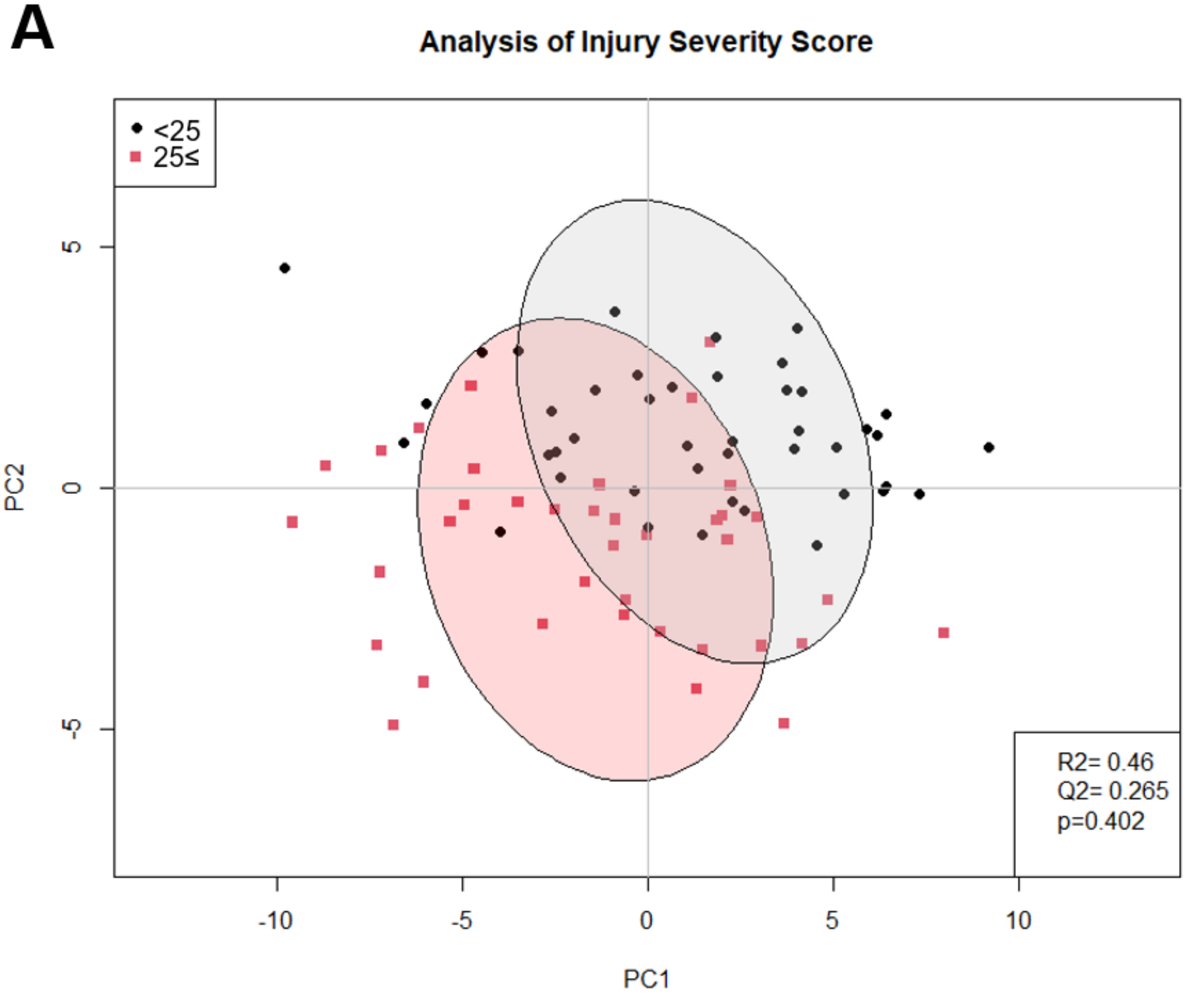

Analysis of Injury Severity Score
<25
25≤
R2= 0.46
Q2= 0.265
p=0.402
PC1
PC2
-10
-5
0
5
10

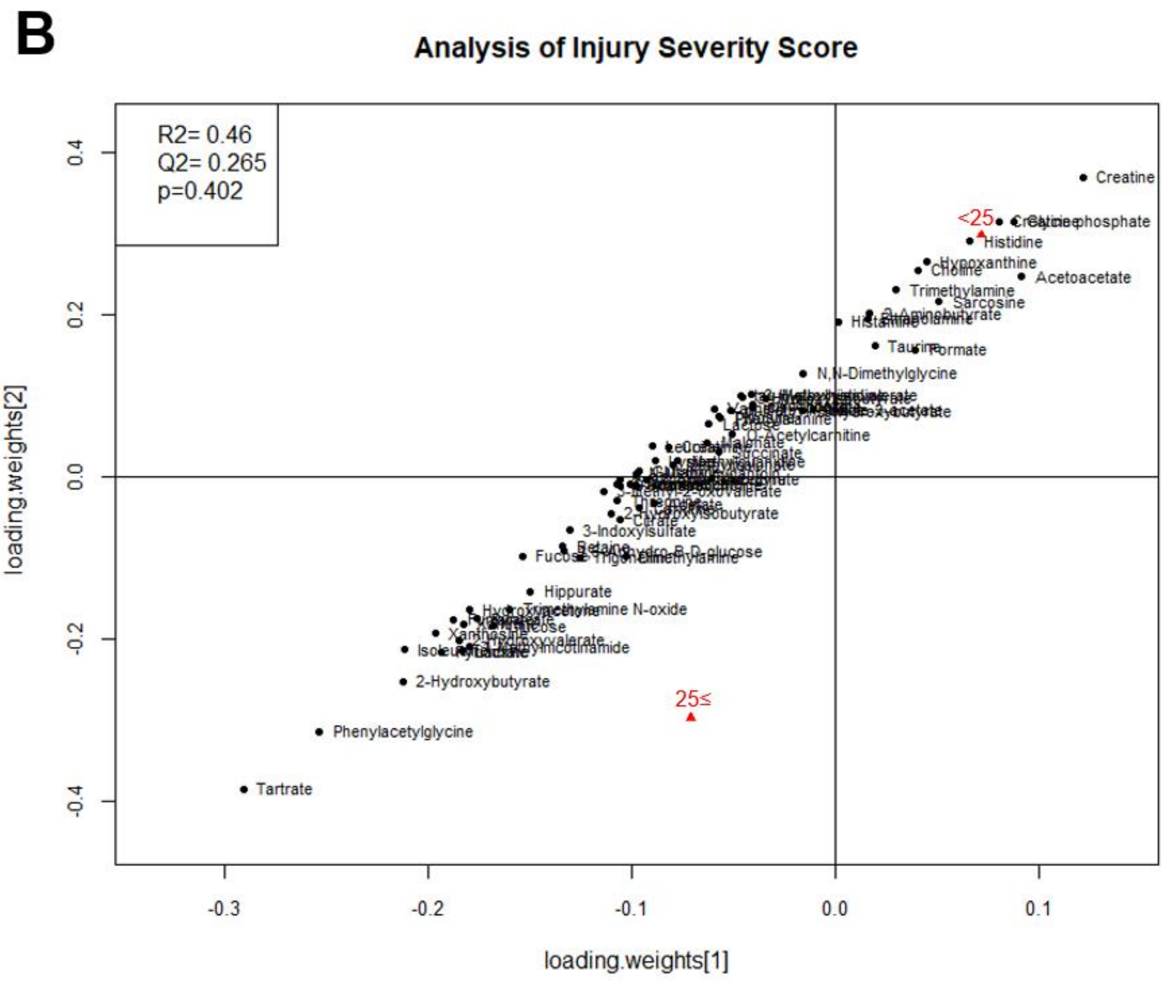

Additional File 3. Analysis for injury severity score (ISS). A) PLS-DA scores plot of urine samples collected from patients who had injury severity scores less than 25 (<25, circle) or greater than or equal to 25 (25≤, square). Each circle and square represents a urine sample. The ellipses represent the 95% confidence interval for the groups. B) Loadings plot for ISS. <25 = patients with an ISS less than 25, 25≤ = patients with an ISS greater than or equal to 25. Loadings show how metabolites contribute to separation seen in the scores plot.
