# Supplementary material for: Urinary metabolites predict mortality or need for renal replacement therapy after combat injury
Source: Crit Care. 2021 Mar 23;25:119. doi: 10.1186/s13054-021-03544-2 (PMC7988986; doi:10.1186/s13054-021-03544-2)
Supplement: Supplementary file 4 — Additional file 4. Analysis for acute kidney injury (AKI) diagnosis. A) PLS-DA scores plot of urine samples collected from patients who were diagnosed with AKI (yes, square) or not diagnoses with AKI (no, circle). Each circle and square represents a urine sample. The ellipses represent the 95% confidence interval for the groups. B) Loadings plot for AKI diagnosis. Loadings show how metabolites contribute to separation seen in the scores plot. [file 13054_2021_3544_MOESM4_ESM.pdf]

A

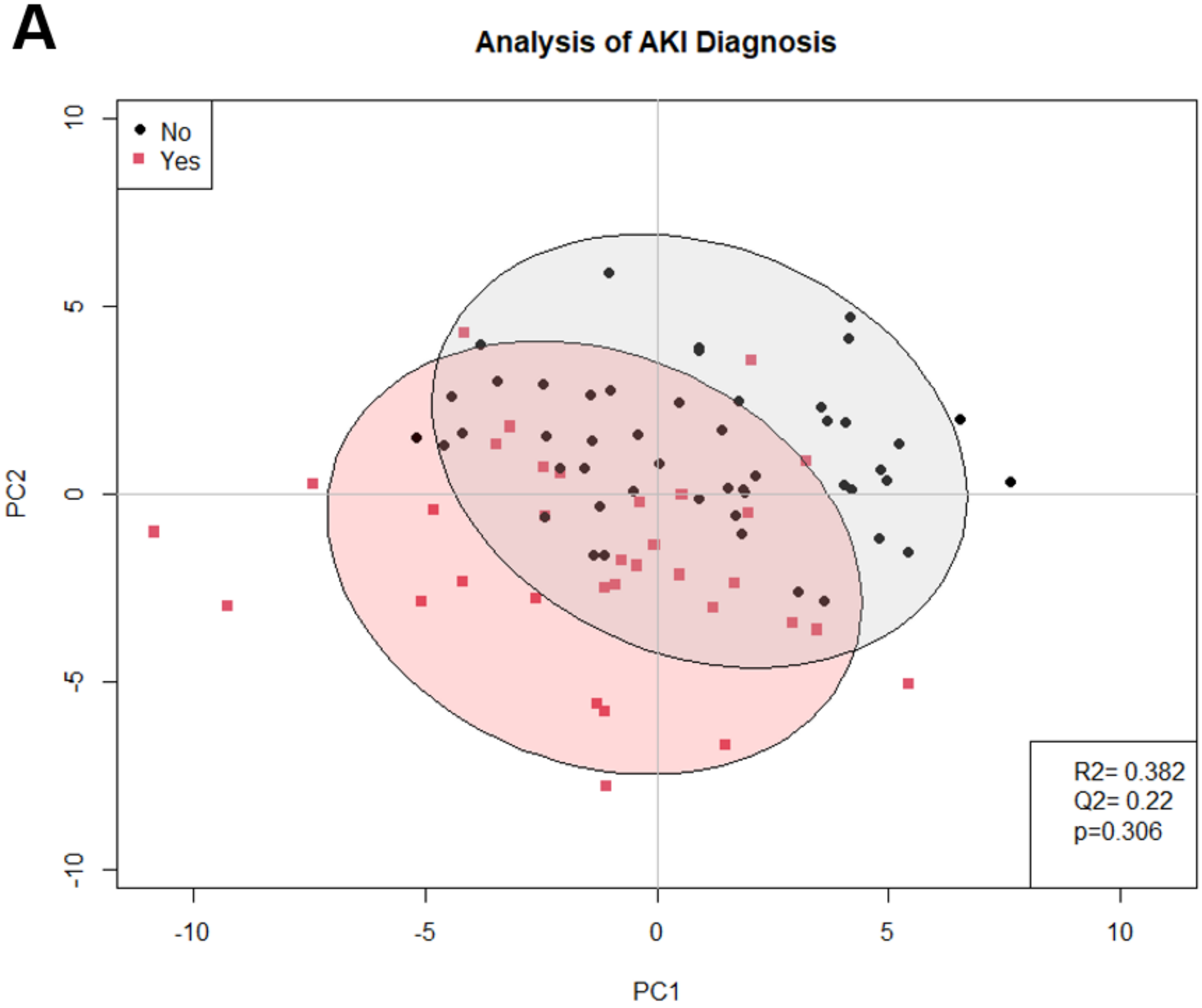

Analysis of AKI Diagnosis
No
Yes
R2= 0.382
Q2= 0.22
p=0.306
PC1
PC2
-10
-5
0
5
10

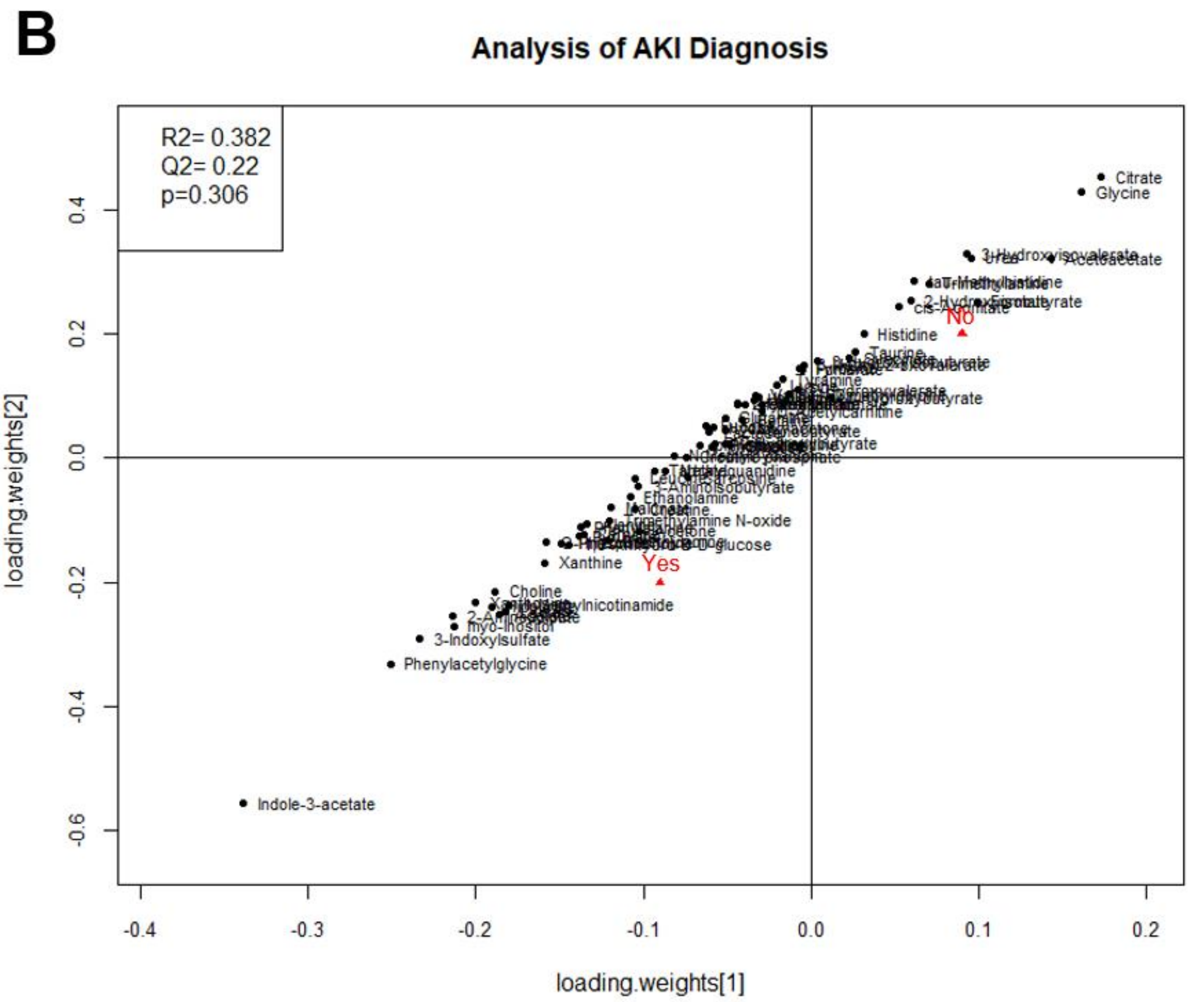

Additional File 4. Analysis for acute kidney injury (AKI) diagnosis. A) PLS-DA scores plot of urine samples collected from patients who were diagnosed with AKI (yes, square) or not diagnoses with AKI (no, circle). Each circle and square represents a urine sample. The ellipses represent the 95% confidence interval for the groups. B) Loadings plot for AKI diagnosis. Loadings show how metabolites contribute to separation seen in the scores plot.
