# Supplementary material for: Urinary metabolites predict mortality or need for renal replacement therapy after combat injury
Source: Crit Care. 2021 Mar 23;25:119. doi: 10.1186/s13054-021-03544-2 (PMC7988986; doi:10.1186/s13054-021-03544-2)
Supplement: Supplementary file 5 — Additional file 5. Boxplots of 1-methylnicotinamide and the relationship to ISS and AKI diagnosis. Metabolite concentrations were normalized by urine output and log-transformed and autoscaled. Boxplots were created using values for the median and interquartile range of each metabolite for each group. A) Boxplot shows 1-methylnicotinamide levels are significantly higher in patients who were diagnosed with AKI compared to those who were not diagnosed with AKI. B) Boxplot shows 1-methylnicotinamide levels are significantly higher in patients who have higher injury severity scores (25≤) versus those with lower injury severity scores (<25). ISS = injury severity score; AKI = acute kidney injury. * = p<0.05. [file 13054_2021_3544_MOESM5_ESM.pdf]

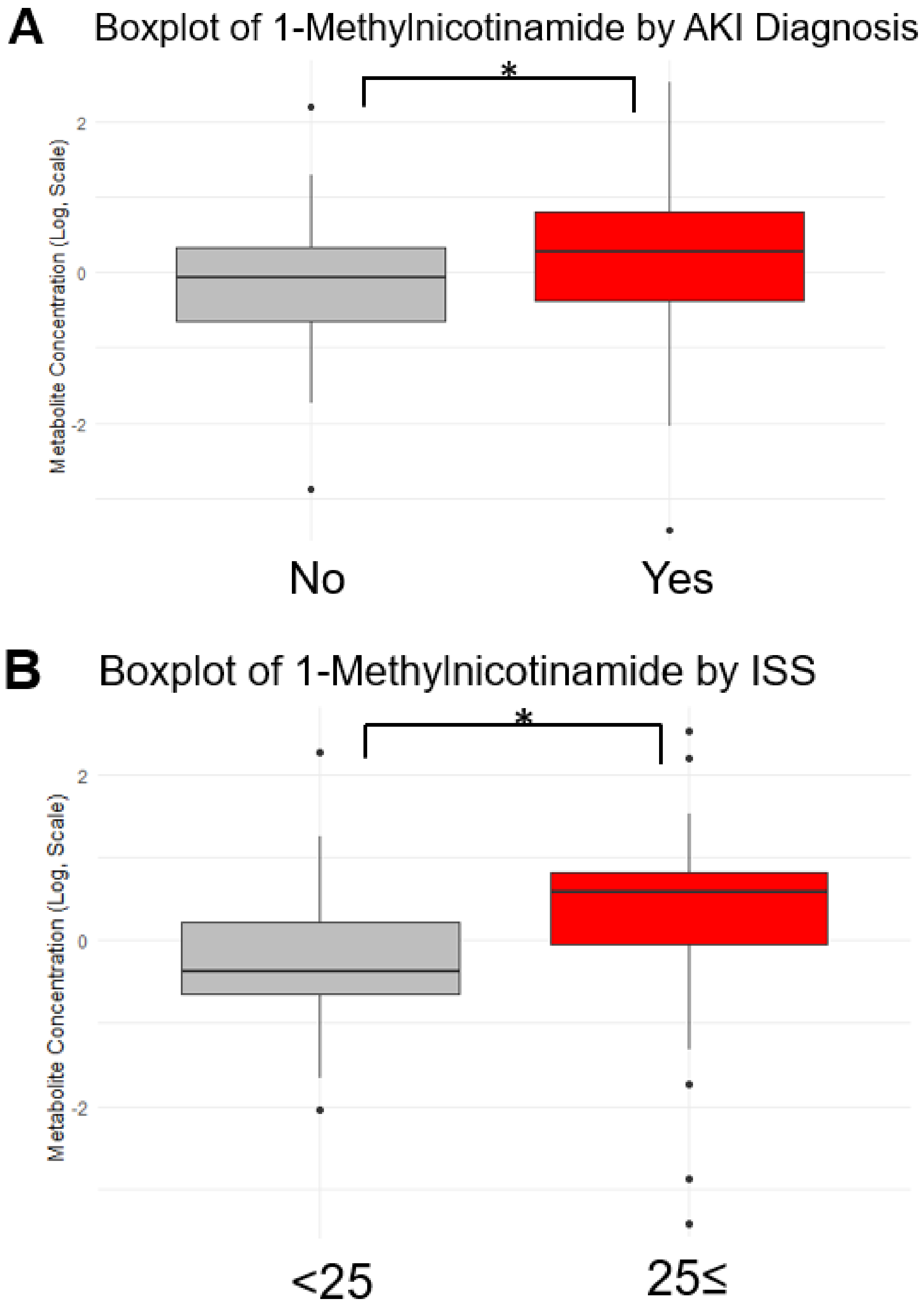

Additional File 5. Boxplots of 1-methylnicotinamide and the relationship to ISS and AKI diagnosis. Metabolite concentrations were normalized by urine output and log-transformed and autoscaled. Boxplots were created using values for the median and interquartile range of each metabolite for each group. A) Boxplot shows 1-methylnicotinamide levels are significantly higher in patients who were diagnosed with AKI compared to those who were not diagnosed with AKI. B) Boxplot shows 1-methylnicotinamide

levels are significantly higher in patients who have higher injury severity scores (25≤) versus those with lower injury severity scores (<25). ISS = injury severity score; AKI = acute kidney injury. * = $p<0.05$.
