# Supplementary material for: Urinary metabolites predict mortality or need for renal replacement therapy after combat injury
Source: Crit Care. 2021 Mar 23;25:119. doi: 10.1186/s13054-021-03544-2 (PMC7988986; doi:10.1186/s13054-021-03544-2)
Supplement: Supplementary file 6 — Additional file 6. Boxplot of 2-hydroxybutyrate and the relationship to mortality/RRT. Boxplots were created using values for the median and interquartile range of each metabolite for each group. Metabolite concentrations were normalized by urine output, log-transformed and autoscaled. Boxplot shows 2-hydroxybutyrate levels are significantly higher in mortality/RRT patients versus patients who survived or did not need RRT. RRT = renal replacement therapy. * = p<0.05. [file 13054_2021_3544_MOESM6_ESM.pdf]

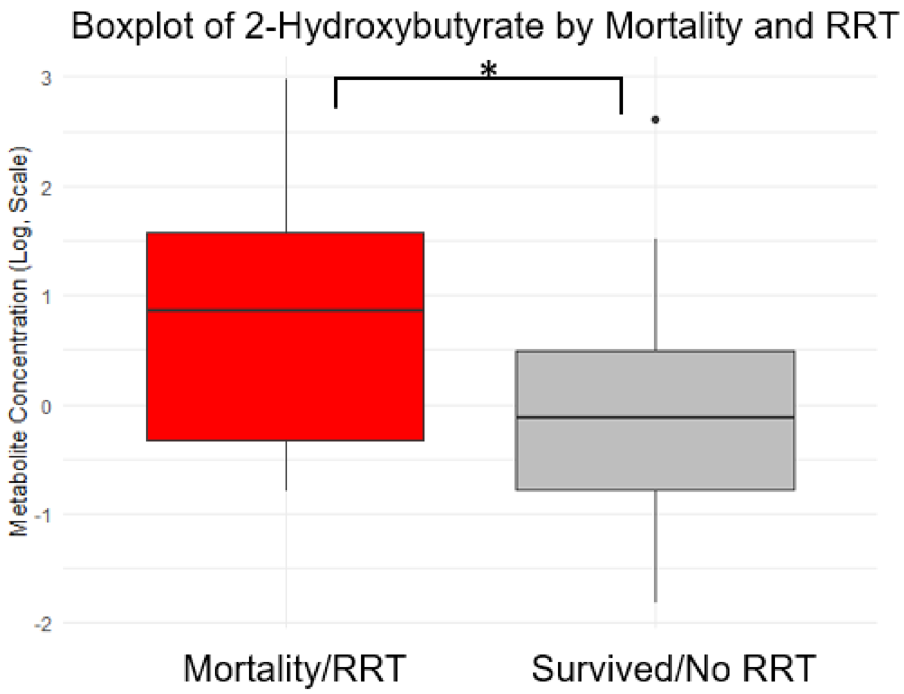

Additional File 6. Boxplot of 2-hydroxybutyrate and the relationship to mortality/RRT. Boxplots were created using values for the median and interquartile range of each metabolite for each group. Metabolite concentrations were normalized by urine output, log-transformed and autoscaled. Boxplot shows 2-hydroxybutyrate levels are significantly higher in mortality/RRT patients versus patients who survived or did not need RRT. RRT = renal replacement therapy. * = $p<0.05$.
